# Structural Attributes Outweigh Topographical Effects in Determining Vascular Plant Diversity Within Southern Apennine Old‐Growth Forests

**DOI:** 10.1002/ece3.74230

**Published:** 2026-09-01

**Authors:** Danilo Travascia, Gianluca Piovesan, Nicodemo Passalacqua, Marco Borghetti, Liliana Bernardo, Sabina Burrascano, Michele Colangelo, Costanza Fiorentino, Domenico Gargano, Antonio Lapolla, Vittoria Marchianò, Anna Rita Rivelli, Aldo Schettino, Francesco Ripullone

**Affiliations:** ^1^ Department of Agricultural, Forest, Food and Environmental Sciences University of Basilicata Potenza Italy; ^2^ Department of Ecological and Biological Sciences (DEB) University of Tuscia, Largo Dell'università Viterbo Italy; ^3^ Department of Biology, Ecology, and Earth Science University of Calabria Arcavacata Italy; ^4^ Department of Plant Biology University of Rome “Sapienza” Rome Italy; ^5^ Pollino National Park Complesso Monumentale Santa Maria Della Consolazione Rotonda Italy

**Keywords:** biodiversity conservation, forest structural complexity, old‐growth forests (OGFs), Southern Apennines, structural heterogeneity index (SHI), vascular plant diversity

## Abstract

Old‐growth forests (OGFs) provide key benchmarks for biodiversity and ecosystem functioning, yet relationships among stand structural heterogeneity, topographic gradients, and multilayered understorey composition remain insufficiently quantified in Mediterranean mountain systems. This study aimed to (i) quantify forest structural heterogeneity in Southern Apennine OGFs, (ii) examine relationships among site conditions, vascular plant diversity and forest structure and (iii) identify the structural attributes most strongly associated with vascular plant species composition while disentangling their contribution from that of topography. Location: Pollino National Park, southern Italy. Structural attributes, topographic variables and vascular plant species composition were recorded across nine candidate OGF sites. After exclusion of one site, the analytical dataset comprised 142 subplots from eight sites. Structural complexity was quantified using a Structural Heterogeneity Index (SHI). Linear mixed‐effects models accounted for subplot nesting within site, while canonical correspondence analysis (CCA), principal coordinate analysis (PCoA) and variation partitioning (VP) assessed structural‐topographic‐floristic relationships. Floristic sampling completeness and sensitivity to the taxon‐frequency threshold were also evaluated. SHI values varied markedly among forest types, with beech and mixed forests exhibiting the highest structural heterogeneity. Across the full multivariate gradient, topographic variables explained 18.8% of variation in structural attributes. In site‐adjusted mixed models, aspect had a significant joint association with SHI, whereas elevation and slope were not significant. Floristic ordination was highly stable across taxon‐frequency thresholds, and pooled sample coverage was 98.65%. VP analysis revealed that the forest‐structure predictor set had a larger unique contribution to floristic variation (23.5%) than topography alone (12.8%), with a substantial shared fraction; large‐tree abundance, canopy cover and decay‐class diversity were among the structural variables significantly associated with vascular plant composition. The ecological complexity of Southern Apennine OGFs reflects an interplay of structural, topographic, historical and floristic dimensions. Integrating stand structure, topographic context and vascular plant composition provides a more robust framework for interpreting and conserving old‐growth forests than any single structural metric.

## Graphical abstract

Forest structure accounted for a larger unique fraction of vascular plant compositional variation in Southern Apennine old‐growth forests (23.5%) than topography alone (12.8%), with a substantial shared contribution between the two predictor sets. Large‐tree abundance, canopy cover and decay‐class diversity were among the structural attributes associated with floristic gradients, highlighting the value of integrating stand structure with topographic context in old‐growth forest assessment and conservation.

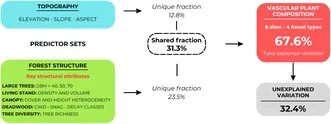

## 1 Introduction

Forest ecosystems are facing unprecedented challenges worldwide, stemming from anthropogenic pressures and climate change, which result in losses of biodiversity and ecosystem services (Pörtner et al. 2021; Pereira et al. 2020). In this context, the scientific understanding and conservation of old‐growth forests (OGFs) have gained increasing international relevance, given their irreplaceable ecological functions as biodiversity reservoirs (O'Brien et al. 2021; Pasques and Munné‐Bosch 2024). OGFs reflect centuries of undisturbed dynamics and are characterised by large and ancient trees, multilayered canopies, abundant coarse woody debris and species‐rich understories (Balloffet and Dumroese 2022). These attributes underpin essential ecological processes such as carbon storage, water regulation and nutrient cycling (Keenan and Read 2012; Gilhen‐Baker et al. 2022). Despite their undeniable ecological and conservation importance, OGFs are extremely rare in Europe, currently covering only about 3% of the continent's forest area due to centuries of intensive land use and logging (Forest Europe 2020; Sabatini et al. 2020; O'Brien et al. 2021). Their limited representation underscores the urgent need to advance strategies for identifying, protecting and monitoring OGFs, as highlighted by the EU Biodiversity Strategy for 2030 (Brumelis et al. 2011; Barredo et al. 2021).

In recent decades, efforts to define and assess OGFs have increasingly relied on structural indicators that reflect forest complexity and ecological integrity. Since the pioneering work of Spies and Franklin (1991), attributes such as tree diameter distributions, deadwood abundance, canopy stratification and biomass accumulation have been widely used to characterise old‐growth structural complexity, including in Mediterranean forests (Badalamenti et al. 2017; Fantini et al. 2020). The structure‐based approach remains the most commonly used framework for identifying and assessing old‐growth status (Storch et al. 2023; Concha et al. 2023; Borghi et al. 2024), often through aggregated indices (Whitman and Hagan 2007; McElhinny et al. 2006; Sabatini et al. 2015). However, the principal methodological challenge is the difficulty of establishing homogeneous and universally applicable structural indicators across forest types and bioclimatic regions, because structural attributes vary naturally with environmental conditions and disturbance history (Ziaco et al. 2012; Burrascano et al. 2013; Gray et al. 2023; Barnett et al. 2023; Bruening et al. 2024). Recent studies therefore emphasise the need to integrate structural indicators with vascular plant composition and environmental context, particularly topography and edaphic factors, to describe the ecological gradients underlying structural variation (van Galen et al. 2018; Jucker et al. 2018; Slezák and Axmanová 2016; Lindenmayer and Bowd 2022). Understorey vascular flora can provide fine‐scale evidence of these gradients and of stand heterogeneity (Giorgini et al. 2015; Filibeck et al. 2015). A key theoretical expectation in OGF ecology is that structural heterogeneity emerges from the interplay between long‐term successional dynamics, stochastic disturbances and site‐level environmental filtering (Franklin et al. 2002; Luyssaert et al. 2008). Under this framework, topography can modulate local microclimate, tree demography and understorey light regimes, while floristic composition reflects the resulting structural–environmental gradients through niche differentiation (Tilman 1994; De Frenne et al. 2021; McNichol et al. 2024). Yet most empirical evidence derives from temperate and boreal systems (Burrascano et al. 2013). Mediterranean mountain OGFs remain underexplored despite pronounced climate seasonality, complex topography and a long history of human land use (Blasi et al. 2010). Recent evidence from Tuscan OGFs showed that structural heterogeneity and old‐growth attributes differ among forest types, with mountain stands generally exhibiting more pronounced old‐growth characteristics than Mediterranean stands (Selvi et al. 2026). Multi‐forest‐type comparisons within a single protected area are therefore valuable for testing whether these patterns also occur in the Southern Apennines and for separating the relative contributions of structure and topography to floristic variation.

Against this background, the objective of this study was to quantify and compare structural heterogeneity, assess the contribution of topographic gradients to structural variation, and identify the structural attributes associated with vascular plant composition across nine candidate OGF sites located within the Pollino National Park. We hypothesised that: (1) structural heterogeneity would differ among Southern Apennine OGF types, providing a regional assessment of patterns recently documented in Tuscan OGFs; (2) elevation and slope would be significantly, but only partially, associated with forest structural variation, leaving substantial variation potentially related to disturbance history and endogenous stand dynamics; and (3) vascular plant composition would be jointly associated with stand structure and topography, with structural attributes accounting for a greater proportion of floristic variation than topography alone.

## 2 Materials and Methods

### 2.1 Study Area

The Pollino National Park (Pollino NP) is the largest protected area in Italy, covering about 190,000 ha in southern Italy (Compagnucci and Mazzoni 2002). Topography is complex, with altitudes ranging from 52 to 2267 m a.s.l. The Park lies in a transitional zone between temperate and Mediterranean climate (Blasi and Michetti 2005), with hot and dry summers at lower altitudes and cold and snowy winters at the highest. Rainfall ranges from 300 mm/year on the Ionian side to 2000 mm/year on the Tyrrhenian side (Todaro et al. 2007). In the inner area, at 2000 m a.s.l., the average winter temperature is −3.2°C, while the average summer temperature is 13.5°C (Colangelo et al. 2021).

Such topographic and climatic variability supports diverse vegetation. Below 800 m a.s.l., Mediterranean scrub dominates, with species such as holm oak ( *Quercus ilex* L.) and downy oak (*Quercus pubescens* Willd.). From 800 to 1400 m a.s.l., forests include turkey oak ( *Quercus cerris* L.), Oriental hornbeam (*Carpinus orientalis* Mill.) and various maple species. Above 1400 m a.s.l., beech forests ( *Fagus sylvatica* L.) are prevalent, sometimes combined with Silver fir ( *Abies alba* ). Close to the tree line, Bosnian pine ( *Pinus heldreichii* Christ var. *leucodermis*) thrives in extreme conditions, occurring as scattered individuals or groups of trees and hosting some of the oldest trees in Europe (Piovesan et al. 2018, 2020; Piovesan, Biondi, Baliva, Dinella, et al. 2019). Despite climate change, these forests have sustained stable growth rates (Colangelo et al. 2021), demonstrating their resilience and conservation value (Baliva et al. 2024).

### 2.2 Old‐Growth Forests Sites

**FIGURE 1:**
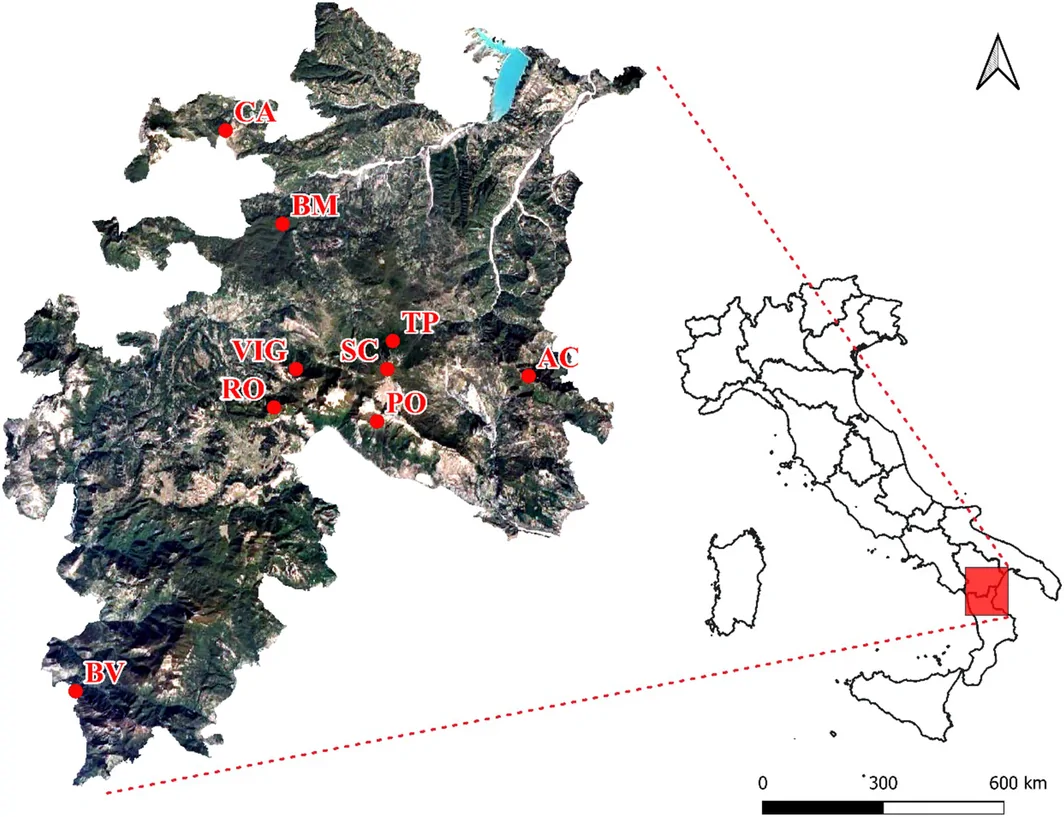
Study areas within the Pollino National Park, Italy. Right: location of the Pollino National Park in Italy. Left: spatial distribution of the nine surveyed candidate OGF stands. AC, Monte Sparviere; BM, Bosco Magnano; BV, Buonvicino; CA, Vaccarizzo; PO, Pollinello; RO, Cozzo Ferriero; SC, Serra di Crispo; TP, Cugno dell'’Acero; VIG, Grattaculo, excluded from the analytical dataset after data screening.

Nine candidate OGF stands were surveyed within the Pollino NP: Buonvicino (BV), Bosco Magnano (BM), Vaccarizzo (CA), Monte Sparviere (AC), Cugno dell'Acero (TP), Cozzo Ferriero (RO), Grattaculo (VIG), Pollinello (PO) and Serra di Crispo (SC) (Figure 1). Candidate stands were preselected within the national old‐growth forest network project and subsequently assessed according to the Italian guidelines, which require at least 60 years without disturbance incompatible with old‐growth status (Castellaneta et al. 2023). During site preselection, compliance with this historical criterion was verified by consulting archival records and management reports available from the Pollino National Park authorities. Selection also considered structural indicators of maturity, including large trees, multilayered canopies and abundant deadwood, and representation of distinct forest types along altitudinal and bioclimatic gradients. The sites encompass a gradient from Mediterranean to montane conditions, including mixed deciduous forests with *Quercus cerris* , *Carpinus orientalis* and *Abies alba* , as well as high‐elevation stands of *Fagus sylvatica* and *Pinus heldreichii* . This range allows testing how structural heterogeneity and vascular plant diversity respond to topography and forest type.

Key attributes—such as elevation, stand composition, living tree volume and maximum age—are summarised in Table S1. Together, the nine surveyed stands provide a representative framework for assessing links among structure, environmental conditions and floristic diversity in highly natural forest ecosystems of southern Italy.

### 2.3 Field Sampling

**FIGURE 2:**
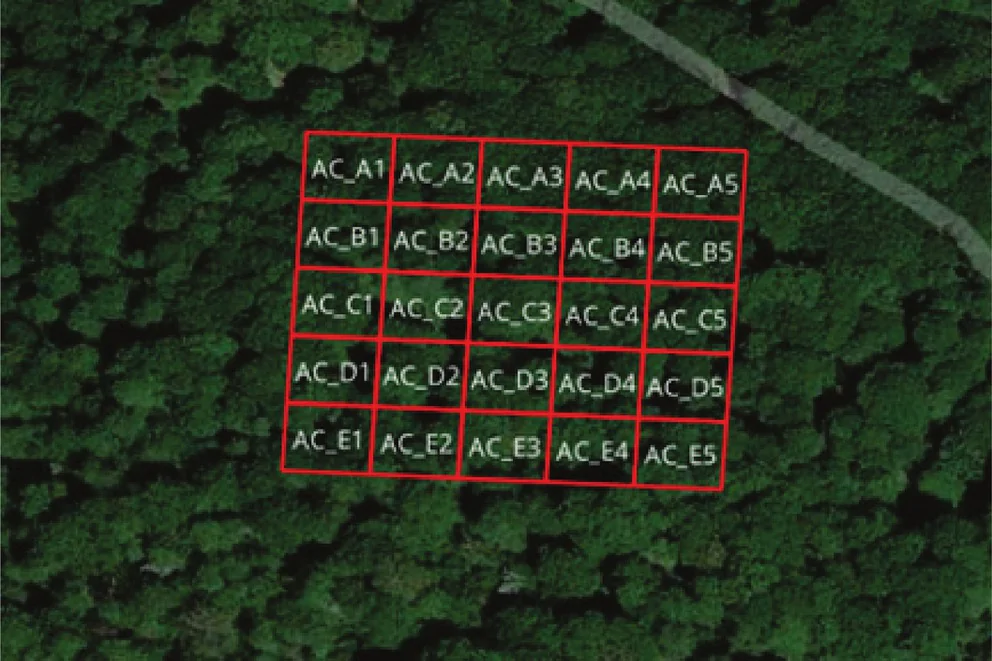
Sampling scheme. Each 1‐ha plot was divided into 25 subplots of 20 × 20 m (400 m^2^), identified by alphanumeric codes and oriented along the site slope.

Within the core area of each site, a 1‐ha plot was established and subdivided into 25 subplots of 400 m^2^ each (Figure 2). Due to topographical constraints (aspect, slope etc.) the sample area in certain forest stands was reduced (CA = 0.36 ha, RO = 0.68 ha, PO = 0.64 ha, BV = 0.40 ha and VIG = 0.64 ha).

Subsequently, vascular plant species composition and abundance were recorded in each subplot. Likewise, geographical coordinates and topographical data, that is, elevation, slope and aspect, were recorded and reported in the UTM 32 N WGS 84 system. Deadwood components, including standing dead trees (SDT), snags, downed dead trees (DDT), coarse woody debris (CWD) and stumps, were measured using different protocols. For SDT, DDT and snags, we measured the tree diameter at breast height (DBH) when greater than 5 cm and height (h) when greater than 130 cm. CWD was recorded for fragments with a diameter greater than 5 cm and a length of over 100 cm. Each fragment was classified according to the decay classes proposed by Hunter (1990).

For stumps, we measured upper diameter (when > 5 cm) and height. Finally, a complete inventory of living trees, shrubs and the regeneration layer was performed using a 5 cm DBH and a 130 cm height threshold. We also recorded tree species, crown features, tree inclination angles and spatial arrangement. Across all investigated OGFs, a total of 168 subplots were surveyed.

## 3 Data Analysis

A total of 24 structural variables, readily obtainable from data collated in forest monitoring programmes (Chirici et al. 2011), were derived (Table S2). Living‐tree, standing dead tree (SDT) and downed dead tree (DDT) volumes were obtained using double‐entry volume equations from the Italian National Forest Inventory. For snags, stumps and CWD, the following equation was used (Equation 1):

(1) $$V=\left(\pi \cdot h/3\right)\;\left(R^{2}+r^{2}+R\cdot r\right)$$

where *V* is the volume, *h* the height, *R* the major radius and *r* the minor radius.

Before analysis, variable distributions and potential outliers were examined. SC was excluded because its structural conditions were markedly outside the range of the remaining stands. Following this, the analysis was restricted to subplots with complete and perfectly matching data on structure, topography and flora, resulting in 142 analytical units spread across eight sites.

### 3.1 Forest Structure Assessment

The Structural Heterogeneity Index (SHI) was derived for each subplot according to the methodology proposed by Sabatini et al. (2015). SHI integrates multiple attributes used to estimate forest structural complexity. A systematic multi‐step process was adopted: (1) Data cleaning: outliers were treated using the interquartile range.

Variables with kurtosis > |2| were excluded, and the remaining variables were reduced through pairwise Pearson correlation (*r* > 0.55). Before exclusion, unsuitable variables were normalised through statistical transformations: living‐tree density (Density_living_tree; Transformed variable: DLT.log), n_dbh_40 (n_dbh_40.log) and quadratic mean diameter (QMD; transformed variable: qmd.log) were log‐transformed; N_dbh (ndbh.sq), n_dbh_50 (n_dbh_50.sq) and dominant height (Dom_height; Transformed variable: Dom_height.sq) were square‐root transformed; necromass volume (Vol_necromass; Transformed variable: nec.tuk) and the dead‐to‐living volume ratio (dlR; Transformed variable: DLR.tu) were adjusted using Tukey transformations. (2) Statistical tests for PCA: selected variables were tested for Principal Component Analysis (PCA) using the Kaiser–Meyer–Olkin and Bartlett tests. (3) PCA implementation: PCA was performed in R (R Core Team 2023) using the FactoMineR (Lê et al. 2008) and factoextra (Kassambara and Mundt 2017) packages. Based on the first three axes, nine attributes were selected: BA_living_tree, dbh_max, H_sd, N_decay_classes, DLT.log, Dom_height.sq., n_dbh_40.log, n_dbh_50.sq. and DLR.tu.

(4) Quartile classification: the nine variables were classified using the 12.5th, 37.5th, 62.5th and 87.5th percentiles, and scores from 0 to 10 were assigned to the resulting classes. A linear regression model was then used to ensure a uniform score distribution. (5) SHI derivation: individual structural‐attribute scores were summed for each subplot and transformed into percentages.

To investigate the relationships between SHI and topography, considering their arrangement within sites, linear mixed‐effects models (LMM) were fitted to the Tukey‐transformed SHI, treating site as a random intercept and standardised altitude, slope, the cosine of aspect and the sine of aspect as fixed effects. Elevation and slope were examined using the partial likelihood ratio test, whilst aspect was assessed jointly by removing both circular components from the overall model. Satterthwaite's tests were then implemented for individual coefficients, whilst the full analysis was repeated using the untransformed SHI as a sensitivity test. Model diagnostic results are shown in Figure S3, whilst the results are presented in Table S7.

### 3.2 Canonical Correspondence Analysis

CCA is a multivariate technique renowned for its effectiveness in revealing complex relationships (Chahouki 2011; Ramette 2007), and was employed here to characterise associations between topographic gradients and forest structural attributes.

Before analysis, the aspect data were transformed by applying the cosine function to derive a ‘northness’ variable, ranging from +1 (north) to −1 (south), and the sine function for the ‘eastness’ variable, also ranging between +1 (east) and −1 (west). All variables were standardised using the ‘*decostand*’ function and ‘range’ method, available in the vegan R package (Oksanen et al. 2024). A correlation matrix was computed to detect and exclude all variables with a Pearson's coefficient greater than 0.60, improving the CCA clustering and model (Tanioka and Yadohisa 2012). The CCA was performed using the ‘*cca*’ function from the vegan package. A Monte Carlo permutation test was performed using 999 random iterations. The model significance was assessed using the ‘*anova.cca’* function, and the topographical variables' contribution was estimated by applying the ‘*anova.term*’ function.

### 3.3 Vascular Plant Species Composition

Vascular plant composition was analysed using PCoA based on Jaccard dissimilarities derived from presence/absence data (McCune and Grace 2002). For the primary ordination, taxa occurring in fewer than four sampling units were excluded from the complete and cleaned herbaceous dataset. Following this filtering step, 151 taxa were retained before cross‐matching between data sources; after restricting the analysis to the final dataset consisting of 142 subplots across eight study sites, 100 taxa were used within the primary ordination matrix. This threshold was applied to reduce potential bias resulting from rare taxa that could significantly influence pairwise dissimilarities and correlations between taxa and axes. PCoA was performed using the ape package (Paradis and Schliep 2019). The first three PCoA axes were correlated with the original taxon occurrences using cor2m in ecodist (Goslee and Urban 2007), and taxa with |*r*| > 0.6 were retained for interpretation (Chytrý et al. 2024). Spearman rank correlations were then used to examine relationships between SHI and the first three PCoA axes. To evaluate the robustness of the chosen minimum frequency threshold, a sensitivity analysis was conducted by repeating the PCoA ordinations with thresholds ranging from one to five. For each threshold, sample configurations and pairwise dissimilarities were compared with those obtained using the reference threshold. Agreement between the ordering structures was assessed using Procrustes correlations, while consistency between the underlying Jaccard dissimilarity matrices was examined using both Pearson and Spearman correlations (Figure S11; Table S8).

Additionally, sampling completeness was evaluated using the unfiltered presence–absence matrix containing 153 taxa across the 142 analytical subplots.

Because the data were incidence‐based, rarefaction and extrapolation, sample‐coverage analyses and the non‐parametric Chao2 richness estimator were calculated using the iNEXT package in R (Chao 1987; Hsieh et al. 2016; Figures S9 and S10; Table S9).

### 3.4 Variation Partitioning Analysis

Variation Partitioning (VP) was performed to quantify the unique and shared contributions of structural and topographic predictor variables to vascular plant composition—as represented by the first three PCoA axes. As a preliminary step, all highly correlated variables (Pearson's *r* > 0.60) were removed, while the retained variables were standardised. Redundancy Analysis (RDA) was then fitted first to the complete model and then to each predictor set to estimate unique and shared fractions. Model and term significance were also evaluated using 999 permutations implemented in the vegan package, whilst Variance Inflation Factors (VIFs) were estimated to assess multicollinearity. Given the nested sampling design, these multivariate analyses were used to characterise associations across the complete sampled gradient and were not interpreted as providing independent subplot‐level causal effects.

## 4 Results

### 4.1 Key Forest Attributes and Structural Complexity

The PCA, performed as part of the SHI, proved essential for identifying key structural attributes, with 62.9% of the total variance accounted for by the first three PCA dimensions. Dimension 1 (23.2%) was linked to tree size and basal area, i.e., BA_living_tree and dbh40.log. Conversely, Dimension 2 (21.0%) was associated with tree height heterogeneity and greater diameter classes (dbh50.sq).

However, additional structural attributes, such as Dom_height.sq. and H_sd, increased the relevance of height‐related metrics in explaining vertical complexity (Figure S1). Finally, Dimension 3 (18.7%) highlighted metrics associated with deadwood volume and decay classes (DLR.tu and N_decay_classes) (Figure S2). These variables fulfilled a large part of the 8 sources of heterogeneity proposed by Sabatini et al. (2015), providing a detailed representation of the forest structure diversity at the considered sites (Table S3).

**FIGURE 3:**
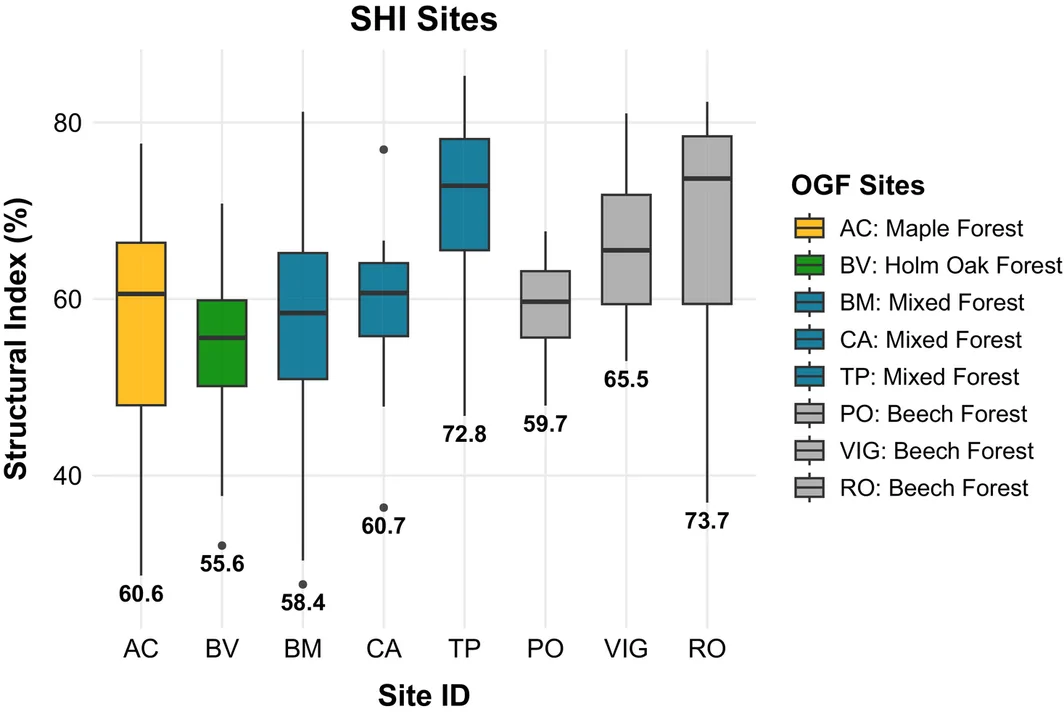
Distribution of SHI values across sites. Whiskers extend to the most extreme observations within 1.5× IQR, boxes represent the 25th–75th percentiles, and horizontal lines indicate medians. Site‐specific means are reported below each box, with colours denoting forest types: AC, Maple (orange); BM/CA/TP, Mixed (blue/light blue); BV, Holm Oak (green); PO/RO/VIG, Beech (grey).

The SHI distribution (Figure 3) shows a considerable variation among the sites. The highest SHI values were found in beech and mixed forests, i.e., RO (73.65%), TP (72.84%) and VIG (65.52%). Conversely, the lowest SHI was observed in the pure holm oak stand BV (55.60%), followed by the mixed‐forest site BM (58.41%).

The LMM, accounting for within‐site clustering, showed no independent association of Elevation (*p* = 0.20) or Slope (*p* = 0.75) with SHI. In contrast, aspect showed a significant joint association with SHI (*p* = 0.006), driven primarily by its cosine component (aspect_cos; *p* = 0.002), which represents the north–south aspect gradient; the sine component (aspect_sin) was not significant (*p* = 0.87). The sensitivity analysis based on untransformed SHI yielded the same overall pattern, with a significant joint association for aspect (*p* = 0.0094) and no significant associations for Elevation or Slope (Figure S3; Table S7).

### 4.2 Topographic Gradients Shape Forest Structural Complexity

**FIGURE 4:**
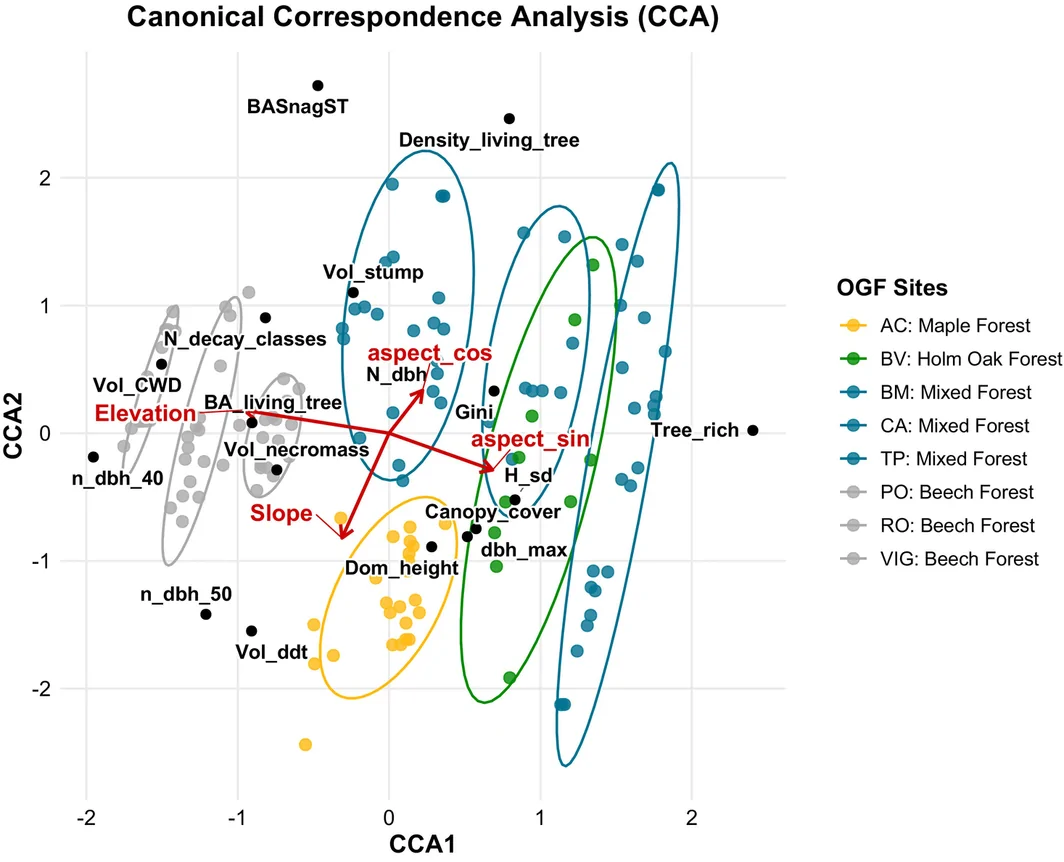
Canonical correspondence analysis (CCA) ordination of forest structural attributes in relation to topographic conditions across the eight analysed candidate old‐growth forest sites in the Pollino National Park. Percentages shown on the CCA axes refer to the proportion of constrained inertia represented by each axis. Black labels represent scores of forest structural variables, whereas red ones represent topographic predictors: elevation, slope, northness (aspect_cos) and eastness (aspect_sin). Coloured points highlight subplot scores, and ellipses enclose 80% of the observations associated with each site under a multivariate normal approximation. Colours indicate the principal forest‐type groups: maple forest (AC), holm oak forest (BV), mixed forests (BM, CA and TP) and beech forests (PO, RO and VIG).

In the CCA, topographic variables accounted for 18.79% of the total variation in structural attributes, whereas 81.21% remained unexplained by the topographic predictor set (Table S4). Across the complete sampled gradient, the constrained CCA including Elevation, Slope, and the cosine and sine components of aspect was significant (*p* = 0.001; Table S5), indicating significant multivariate associations between topographic gradients and forest structural variation (Figure 4).

Relevant attributes, such as coarse woody debris volume (Vol_CWD), number of decay classes (N_decay_classes), necromass volume (Vol_necromass) and basal area of living trees (BA_living_tree), frequently employed in the OGF's definition, were mainly located along the negative section of the CCA1 axis. Such attributes, representative of mature and undisturbed forest ecosystems, were oriented towards PO, RO and VIG sites, characterised by greater elevation and slope.

In contrast, attributes such as tree richness (Tree_rich), maximum diameter at breast height (dbh_max), canopy cover (Canopy_cover), height standard deviation (H_sd) and Gini coefficient (based on basal area of diameter classes; Gini) were positively correlated with the CCA1 axis and related to sites at lower altitude and slope, such as CA, BV and BM. Additional attributes, such as Vol_stump, BA_snagST and N_dbh, located in the upper quadrant, appeared to be related, instead, to the TP site. The overall constrained CCA model was significant (*p* = 0.001; Table S5). Among the individual topographic terms, Elevation showed the largest contribution, with the highest chi‐squared value (0.05719) and *F*‐ratio (18.6466), followed by Slope (chi‐squared = 0.02150, *F* = 7.0087); both terms were significant at *p* = 0.001 (Table 1). Both aspect components were also significant in the term‐wise tests, although the contribution of aspect_cos was smaller (aspect_cos: *F* = 2.1302, *p* = 0.028; aspect_sin: *F* = 4.1384, *p* = 0.001).

**TABLE 1:** Term‐wise significance of topographic variables in the canonical correspondence analysis (CCA) of forest structural attributes.

Permutation test for CCA under reduced model (Terms)				
Number of permutations: 999				
	df	Chi square	*F*	Pr(>*F*)
Elevation	1	0.05719	18.6466	0.001***
Slope	1	0.02150	7.0087	0.001***
aspect_cos	1	0.00653	2.1302	0.028*
aspect_sin	1	0.01269	4.1384	0.001***
Residual	138	0.42325		

*Note:* Significance levels: *p < 0.05; ***p < 0.001.

### 4.3 Vascular Plant Compositional Patterns Across OGFs

**FIGURE 5:**
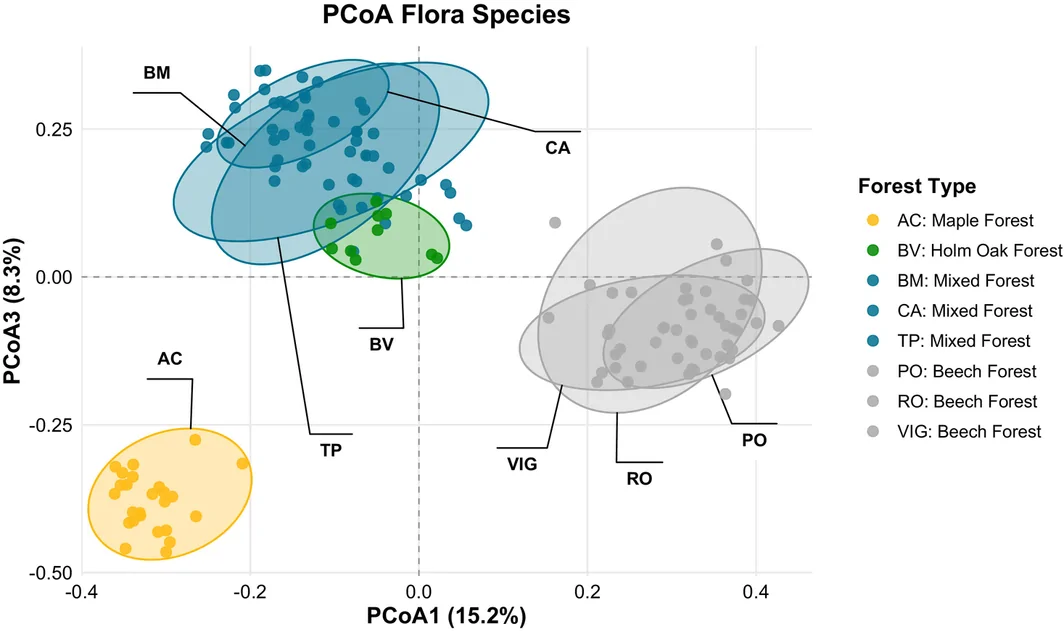
PCoA ordination based on Jaccard dissimilarities in vascular plant composition across the analysed OGF sites. The first two axes explained approximately 28.5% of total variation. Each point represented an OGF subplot, marked and grouped by ellipses according to their site.

The PCoA analysis, based on Jaccard dissimilarities, explained approximately 37.0% of the total variation along its first three axes (PCoA1 = 15.22%; PCoA2 = 13.40%; PCoA3 = 8.37%), with the first two axes together accounting for 28.62% of the variance. The PCoA biplot (Figure 5) also revealed three broad ordination groupings: the beech forest sites (PO, RO and VIG) in the centre‐right region; mixed forest sites (BM, CA and TP), together with the holm oak site (BV), in the top‐left region; and the maple forest site (AC) in the bottom‐left region.

Species typical of temperate European deciduous forests were commonly found across the examined sites. Nine species, including *Polystichum setiferum*, *Lactuca muralis* , *Aremonia agrimonioides* and *Daphne laureola* , were shared across all three ordination groupings, while 18 species, including *Sanicula europaea*, *Galium odoratum* , *Viola riviniana*, *Cardamine chelidonia* and *Clinopodium grandiflorum*, were found exclusively within stands dominated by *Fagus sylvatica* , indicating floristic affinity between the mixed‐forest and beech‐forest sites.

In contrast, the differentiation between beech forests and mixed forests was associated with the presence of microthermic species in the former (e.g., *Adenostyles australis*, *Asyneuma trichocalycinum*, *Senecio squalidus* and *Carduus affinis*) and mesophilous macrothermic species in the latter (e.g., *Ilex aquifolium* , *Ruscus aculeatus*, *Hedera helix* and *Cyclamen hederifolium* ). Notably, the species characterising beech forests were biogeographically relevant, being endemic and/or South‐European orophilous species (Di Pietro 2009; Dillenberger and Kadereit 2013; Peruzzi et al. 2015).

AC was distinguished by the presence of eutrophic/nitrophilous species, such as *Alliaria petiolata, Arctium lappa* and *Urtica dioica* . Its floristic affinities with mixed or beech forests were partly mediated by eutrophic taxa, such as *Geum urbanum* , *Rumex sanguineum* and *Arum cylindraceum*, with the former and *Hordelymus europaeus*, *Elymus caninus* , *Galium aparine* , *Anthriscus nemorosa*, *Epilobium montanum* and *Ranunculus brutius* with the latter.

Among the taxa retained for interpretation (|*r*| > 0.6), PCoA1 was positively associated with *Adenostyles australis*, *Asyneuma trichocalycinum*, *Senecio squalidus* , *Galium odoratum* and *Clinopodium grandiflorum*, whereas negative associations were observed for *Arum cylindraceum*, *Fraxinus excelsior* and *Urtica dioica* . PCoA2 was positively associated with *Abies alba* , *Quercus cerris* , *Ilex aquifolium* and *Ruscus aculeatus*, and negatively associated with *Doronicum orientale* , *Elymus caninus* , *Fraxinus excelsior* , *Galium aparine* , *Hordelymus europaeus*, *Poa nemoralis* and *Ranunculus brutius*. PCoA3 showed negative associations with *Abies alba* , *Cardamine chelidonia* and *Viola riviniana* (Figure S4). These taxon–axis correlations describe species associations with the principal compositional gradients and were not interpreted as direct measurements of soil, microclimatic or disturbance conditions. Spearman's correlation analysis between the PCoA axes and SHI values provided additional insights. PCoA1 was not significantly associated with SHI (Spearman's *ρ* = −0.051, *p* = 0.5495; Figure S5).

In contrast, PCoA2 showed a positive association with SHI (*ρ* = 0.333, *p* = 5.28 × 10^−5^; Figure S6), while PCoA3 showed a weaker positive association (*ρ* = 0.185, *p* = 0.0276; Figure S7). Together, these results indicate that forest structural heterogeneity was associated with selected, but not all, components of vascular plant compositional variation. Sampling completeness of the unfiltered floristic matrix was high at the pooled level, with a sample coverage of 0.9865 and a Chao2 asymptotic richness estimate of 170.5 taxa (95% CI: 159.4–200.6). Site‐level coverage was more variable, particularly at sites represented by fewer analytical subplots (Figures S9 and S10; Table S9). Sensitivity analyses further confirmed that the primary PCoA ordination was highly stable with respect to the taxon‐incidence filtering criterion. Across minimum occurrence thresholds ranging from one to five, Procrustes correlations with the baseline ordination (threshold = 4) ranged from 0.9992 to 0.9997, while Pearson and Spearman matrix correlations between pairs of Jaccard dissimilarities ranged from 0.9954–0.9980 and 0.9970–0.9989, respectively (Figures S9–S11; Tables S8 and S9).

### 4.4 The Role of Forest Structure and Topography in Shaping the Vascular Plant Species Distribution

The VP analysis (Figure S8; Table 2) showed that forest structure and topography jointly accounted for 67.6% of the variance in vascular plant composition. The shared fraction accounted for 31.3%, while the unique contribution of structural attributes was 23.5%, exceeding the unique contribution of topography alone (12.8%). However, the remaining 32.4% of unexplained variance suggested that additional unmeasured drivers, including biotic interactions, historical land use, soil properties and fine‐scale environmental heterogeneity, may also contribute to the observed floristic patterns.

**TABLE 2:** Partitioning of variance in vascular plant composition explained by (X1) forest structure and (X2) topography (full RDA model). Reported values include adjusted *R* ^2^, individual fractions, and the percentage of variance explained by pure effects, shared effects and residuals.

Partition of variance in RDA				
Fraction	Description	Adj. *R* ^2^	Individual fraction	Explained variance (%)
X1	Forest structure effect	0.54834	0.23538	23.5
X2	Topography effect	0.44102	0.12806	12.8
X1 + X2	Shared effect	0.67640	0.31296	31.3
Residuals			0.32360	32.4

**FIGURE 6:**
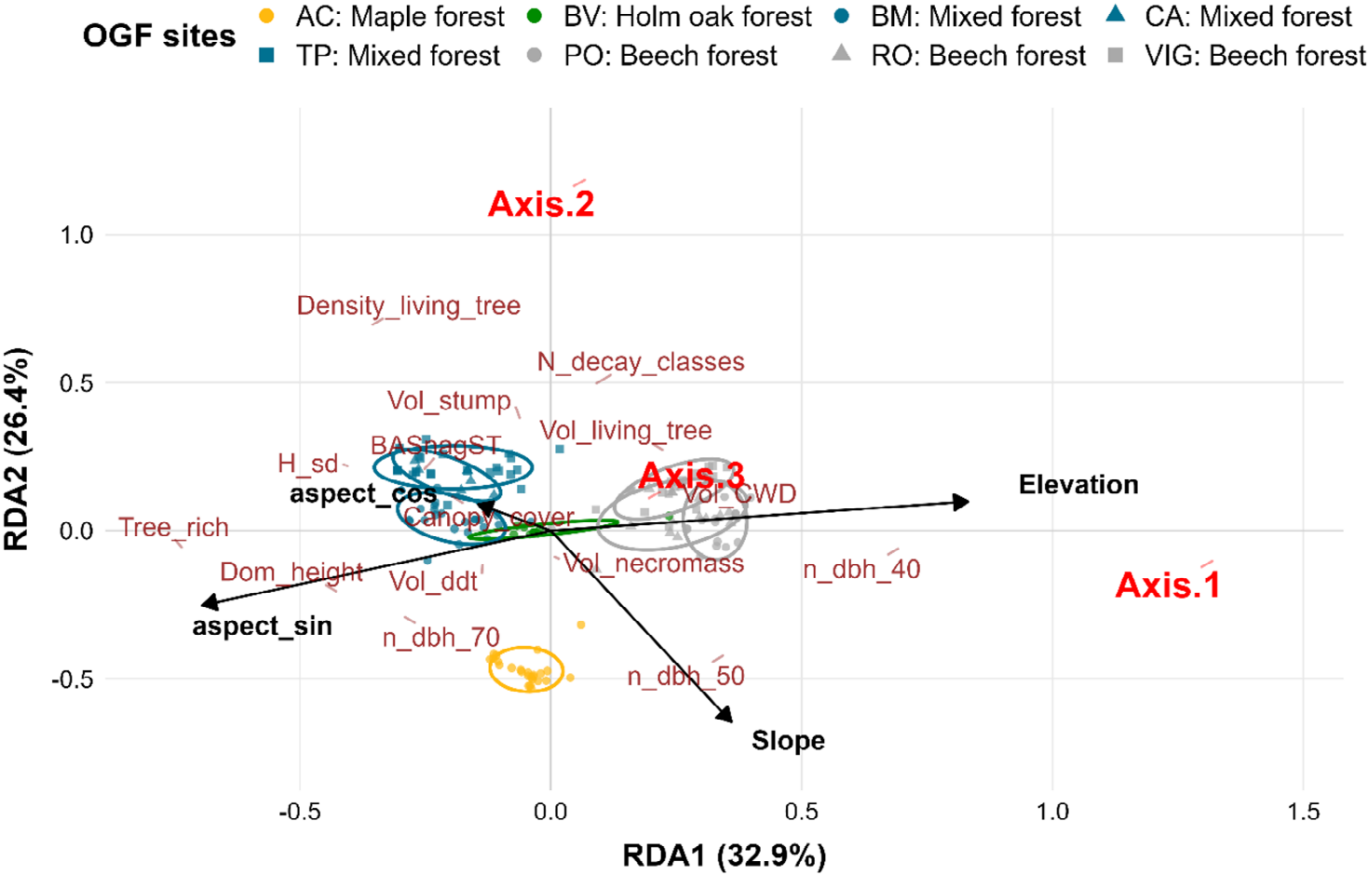
RDA biplot relating vascular plant compositional gradients to forest structural and topographic predictors. Vascular plant composition is represented by the first three PCoA axes (Axis 1, Axis 2, Axis 3) used as response variables in the RDA, while forest structural and topographic variables constitute the explanatory predictor set. OGF sites are coloured by forest type.

The complete RDA model was significant (*p* = 0.001; Table S6), supporting an overall association between vascular plant composition and the combined set of structural and topographic factors. Figure 6 shows the RDA ordination plot, which provides a visual representation of these relationships.

Axis 1 (RDA1 = 32.9%) primarily reflected topographic gradients, being positively associated with elevation, slope and the abundance of trees with DBH > 40 cm (n_dbh_40). Conversely, it showed negative relationships with tree species richness, dominant height (Dom_height) and eastness (aspect_sin). Axis 2 (RDA2 = 26.4%), on the other hand, captured variation in different stand structural attributes.

Indeed, this axis was positively associated with tree density (Density_living_tree), canopy cover (Canopy_cover) and number of decay classes (N_decay_classes), while showing a negative association with tree species richness (Tree_rich).

Sequential permutation tests by term within the complete RDA model identified significant contributions from Basal area of snag and standing dead trees (BASnagST), canopy cover (Canopy_cover), number and volume of living trees (Density_living_tree; Vol_living_trees), volume of coarse woody debris (Vol_CWD), number of decay classes (N_decay_classes), number of trees with DBH > 40, 50 and 70 cm (n_dbh_40; n_dbh_50; n_dbh_70), tree species richness (Tree_rich) and height heterogeneity (H_sd; Table 3). Elevation (Elevation) and slope (Slope) were also significant (*p* = 0.001), while eastness (aspect_sin) showed a weaker but still significant contribution.

**TABLE 3:** Permutation test results for individual forest structural and topographical variables in the RDA model.

Permutation test for RDA under reduced model (by Term)					
Number of permutations: 999					
Variable	Abbreviation	df	Variance	*F*	Pr(>*F*)
Basal area of snags and standing dead trees	BASnagST	1	0.019282	38.82	0.001***
Canopy cover	Canopy_cover	1	0.007775	15.65	0.001***
Volume of coarse woody debris	Vol_CWD	1	0.010552	21.24	0.001***
No. of living tree/ha	Density_living_tree	1	0.028985	58.35	0.001***
Volume of necromass	Vol_necromass	1	0.000936	1.88	0.129
Volume of living trees	Vol_living_trees	1	0.007322	14.74	0.001***
No. of decay classes	N_decay_classes	1	0.008133	16.37	0.001***
No. of tree with DBH > 40	n_dbh_40	1	0.018069	36.37	0.001***
No. of tree with DBH > 50	n_dbh_50	1	0.008576	17.26	0.001***
No. of tree with DBH > 70	n_dbh_70	1	0.003503	7.05	0.001***
No. of tree species	Tree_rich	1	0.008032	16.17	0.001***
Volume of stump	Vol_stump	1	0.000856	1.72	0.144
Volume of downed dead trees	Vol_ddt	1	0.000620	1.25	0.282
Dominant height	Dom_height	1	0.001329	2.68	0.061
Height standard deviation	H_sd	1	0.005115	10.30	0.001***
Elevation	Elevation	1	0.019233	38.72	0.001***
Slope	Slope	1	0.004636	9.33	0.001***
Aspect (North–South)	aspect_cos	1	0.001195	2.40	0.066
Aspect (East–West)	aspect_sin	1	0.001692	3.41	0.019*
Residual		122	0.060603		

*Note:* Significance levels: *p < 0.05; ***p < 0.001.

Conversely, northness (aspect_cos), dominant height (Dom_height), necromass volume (Vol_necromass), stump volume (Vol_stump) and downed dead‐tree volume (Vol_ddt) were not significant.

**FIGURE 7:**
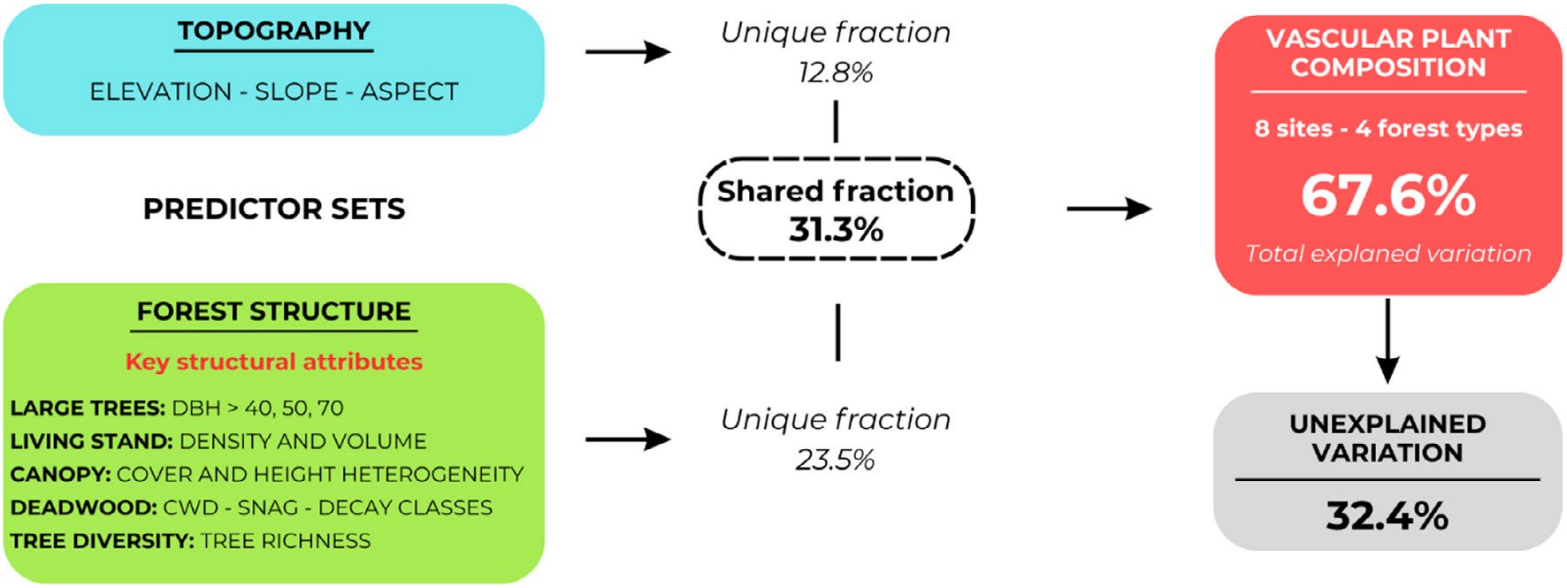
Conceptual synthesis of the main associations between topographic and forest structural predictors and vascular plant community composition. The two predictor sets include: (a) *topography* (elevation, slope and aspect) and (b) *forest structure* (large‐tree abundance represented by DBH > 40, 50, and 70 cm; living‐tree density and volume; canopy cover and height heterogeneity; coarse woody debris, snags and decay‐class diversity; and tree species richness). Percentages represent the fractions of variation in vascular plant composition obtained from variation partitioning based on redundancy analysis (RDA). Topography uniquely accounted for 12.8% of floristic variation, forest structure uniquely accounted for 23.5%, and the shared fraction between the two predictor sets accounted for 31.3%. The full model explained 67.6% of the variation, leaving 32.4% unexplained.

Overall, the RDA indicated that differentiation among the four forest type groups was associated with both topographic gradients and forest structural attributes. Consistent with our hypothesis, forest structure contributed a greater unique fraction of floristic variance than topography alone. Living‐tree density, canopy cover, large‐tree density, height heterogeneity and deadwood‐related attributes—including coarse woody debris volume and decay‐class diversity—were among the principal structural variables associated with vascular plant composition. Figure 7 provides a conceptual overview that integrates the main structural and topographical relationships with the results from the VP analysis.

## 5 Discussion

This study examined nine potential old‐growth forests (OGFs) within the Pollino National Park, using integrated analyses carried out on 142 subplots from eight sites. Our results provide insight into the relationships between topographic context, stand structure and vascular plant composition of the Southern Apennines' OGFs. Along the full gradient, structural attributes accounted for a greater unique fraction of floristic variation than topography alone, although a substantial shared component was also observed.

The residual unexplained variation pointed to the involvement of additional factors, such as historical disturbances, local stand dynamics, soil properties and fine‐scale microclimatic heterogeneity.

### 5.1 Forest Structural Heterogeneity as a Function of Topographic Gradients

Our multivariate analysis indicated a significant but partial association between topography and forest structural heterogeneity. In the structural CCA, the topographic predictor set—elevation, slope and aspect—collectively accounted for 18.8% of the variation in structural attributes across the complete sampled gradient. Thus, topography contributed to structural differentiation among stands but accounted for only a limited fraction of the observed heterogeneity. The marked differences in structural heterogeneity observed among the Southern Apennine stands are broadly consistent with forest‐type‐related patterns recently documented in Tuscan OGFs (Selvi et al. 2026), extending this emerging evidence to a distinct biogeographic and environmental context.

Topography may also have contributed indirectly to the persistence of old‐growth attributes by limiting historical accessibility and timber extraction.

Steep, rugged, and inaccessible terrain can reduce the feasibility of logging, potentially promoting the retention of structural features associated with late‐stage succession (Ziaco et al. 2012; Castellaneta et al. 2023).

The high SHI values, abundant coarse woody debris, and diverse decay classes observed at remote sites such as RO and VIG are consistent with this interpretation, although the present observational data cannot disentangle historical accessibility effects from other site‐specific environmental and land‐use factors.

The mixed‐effects analysis added a scale‐dependent qualification to this multivariate pattern. After accounting for within‐site clustering, elevation and slope showed no independent association with subplot‐level SHI, whereas aspect—tested jointly via its cosine and sine components—was significant. Thus, the CCA captures structural covariation across the complete among‐ and within‐site gradient, whereas the mixed models isolate topographic effects after controlling for Site. The large residual variance in the CCA (81.2%) and the non‐significant elevation and slope effects in the LMM both point to the substantial influence of additional drivers—including soil fertility, disturbance history, stand development and local microclimate—in shaping structural heterogeneity.

Height and diameter class distributions, together with maximum diameter at breast height, revealed strong vertical and horizontal heterogeneity, which may support biodiversity (Pickering et al. 2024). Given this structural complexity, the high standing‐dead‐tree volume and canopy height observed at site AC likely reflect the legacy of recurrent natural disturbances that affect taller trees (Jackson et al. 2024). These patterns align with the successional trajectories proposed by Oliver and Larson (1996) and Franklin et al. (2002), in which disturbance events and subsequent stand development generate the structural heterogeneity typically found in old‐growth forests.

### 5.2 Stand Structure and Topography as Primary Drivers of Vascular Plant Composition

Vascular plant species composition was associated with forest structure and topography. The PCoA showed three broad compositional groupings: the beech‐forest sites; the mixed‐forest sites together with the holm oak site BV; and the maple‐dominated site AC. Their differentiation was subsequently associated with structural and topographic predictors in the RDA. For instance, *Adenostyles australis*, *Asyneuma trichocalycinum*, *Senecio squalidus* and *Carduus affinis*, typically occurring in cold, mountain environments, underscore the ecological distinctiveness of high‐altitude beech forests. Furthermore, the occurrence of certain species, some of which are endemic or restricted to Southern European mountains, highlights the biogeographical significance of these communities. Conversely, species such as *Alliaria petiolata* , *Arctium lappa* and *Urtica dioica* are characteristic of eutrophic and nitrophilous environments, often associated with recurring disturbance processes (Matuszkiewicz et al. 2024). Among the sites, BV and AC exhibited particularly interesting floristic patterns. BV, a pure low‐elevation holm oak forest, showed an unexpected similarity in vascular plant composition to mixed forests dominated by beech, silver fir or Turkey oak. This similarity may reflect its position on slopes facing the Tyrrhenian Sea, where milder climatic conditions allow holm oak to extend to higher elevations, facilitating contact with mountain forest types. In contrast, AC, a maple‐dominated stand, displayed a distinct vascular plant assemblage, distinguished by *Alliaria petiolata* , *Arctium lappa* and *Urtica dioica* , which may indicate local grazing disturbance. The occurrence of these contrasting taxa is consistent with the broader compositional differentiation observed among sites and may reflect differences in their environmental and disturbance context.

Importantly, these compositional patterns were highly robust to the treatment of low‐frequency taxa. Repeating the PCoA across minimum incidence thresholds ranging from one to five produced highly concordant sample configurations and Jaccard dissimilarity structures. Sampling completeness was evaluated separately using the unfiltered 153‐taxon matrix. Although pooled sample coverage was high, Chao2 estimates, together with the lower coverage observed at some individual sites, indicate that additional rare taxa may have remained undetected. Accordingly, our conclusions concern the dominant compositional gradients and their associations with forest structure and topography rather than an exhaustive characterisation of the rare vascular plant component.

### 5.3 Linking Structural Heterogeneity and Topographic Setting to Understorey Floristics and Late‐Successional Forest Signatures

Variation partitioning provided a complementary structural perspective on these floristic patterns. The forest‐structure predictor set accounted for a larger unique fraction of vascular plant compositional variation (23.5%) than topography alone (12.8%), indicating that structural attributes represented an important component of the habitat heterogeneity associated with floristic differentiation. Large and old trees can stabilise forest microclimate and provide suitable conditions for specialised vascular plants (Lindenmayer 2017). Likewise, the accumulation of late‐successional features—including large trees, high canopy cover and multiple deadwood decay stages—can increase microhabitat diversity and microclimatic buffering, thereby providing a plausible ecological context for the structural–floristic associations observed here (Franklin et al. 2002; Tilman 1994).

Canopy complexity was among the structural attributes associated with understorey plant composition, consistent with its ecological role in modulating light availability and microclimatic conditions, which are also important for *Abies alba* regeneration in Mediterranean mountain environments (Ripullone et al. 2016). The number of decay classes further emphasised the ecological significance of deadwood decomposition phases, which can enhance substrate heterogeneity and provide habitats for saproxylic organisms and herbaceous species (Graf et al. 2022). Tree species richness and coarse woody debris volume were likewise significantly associated with vascular plant compositional variation in the RDA, consistent with broader evidence linking forest structural complexity with floristic diversity. Ecologically, greater canopy and tree‐species diversity can increase niche availability, while coarse woody debris provides substrates and resources for decomposer communities and vascular plants (Chećko et al. 2015). More generally, structurally complex stands can provide a wider range of microhabitats and buffer environmental variability, potentially contributing to greater ecological resilience (Ontl et al. 2020). Site‐specific patterns provide further evidence of the structural–floristic linkage. At TP, the basal area of snags and standing dead trees, together with the number of diameter classes, was consistent with a structurally heterogeneous stand shaped by past disturbance and subsequent stand development. Such features are commonly associated with late‐successional dynamics and ecological continuity in mature forests (Piovesan, Biondi, Baliva, De Vivo, et al. 2019). They may therefore serve as useful indicators of natural forest development and help assess structural maturity across forest types and biogeographic regions.

Taken together, the structural and floristic attributes observed in this study reinforce the conservation relevance of structurally complex old‐growth stands. Our findings are consistent with previous studies emphasising the importance of large trees, deadwood and vertical heterogeneity for biodiversity maintenance (Bauhus et al. 2009; Müller and Bütler 2010). In the Mediterranean mountain forests examined here, large old trees, coarse woody debris, canopy layering and decay‐stage diversity emerged as particularly relevant structural components associated with habitat heterogeneity and vascular plant composition. These attributes should therefore be considered alongside edaphic, topographic and climatic variables in conservation planning. Structural complexity has also been associated with biodiversity across multiple taxonomic groups, including birds, bats, and understorey plants, further supporting the retention of structural legacies in managed and protected forests (Govedar et al. 2024). The conservation of large trees may additionally provide important ecosystem benefits through their disproportionate contribution to forest carbon storage (Lutz et al. 2018).

These principles may also be relevant beyond the Southern Apennines, but their transferability requires validation against local forest histories and ecological conditions. Likewise, the possibility that some present‐day shrublands formerly supported structurally complex forests represents an important historical‐ecological hypothesis, but it cannot be inferred from the present data and requires palaeoecological, historical or long‐term land‐use evidence. Effective conservation planning should therefore integrate physical setting, structural legacies, biodiversity targets and socio‐economic context, recognising that old‐growth attributes reflect not only topographic and climatic constraints but also past land use and disturbance regimes.

### 5.4 Study Limitations and Opportunities

Although the SHI captured marked structural differences among the Southern Apennine OGFs, its interpretation remains context‐dependent. This is particularly evident at PO, a site recognised for its high degree of naturalness (Piovesan et al. 2020) but characterised by a relatively low SHI. Where topographical constraints limit canopy height and tree growth, the index may therefore underestimate ecological maturity by confounding structural expression with site‐specific structural potential. Consequently, while SHI is useful for comparative assessments within this regional gradient of forest types, broader application across different biomes would require recalibration of variable selection and thresholds according to local structural and environmental constraints. A further methodological consideration concerns the nested sampling design. The 1‐ha plots, subdivided into 400m^2^ subplots, followed the Italian Environmental Ministry protocol for OGF‐characterisation (Chirici et al. 2011); however, subplots within a site share environmental and historical conditions, creating potential pseudoreplication if treated as fully independent (Hurlbert 1984). We addressed this dependence in the univariate SHI–topography analyses by fitting mixed‐effects models with Site as a random intercept.

The primary variation partitioning describes associations across the complete sampled gradient and should therefore be interpreted at the system level rather than as evidence of independent causal effects at the subplot scale. Future studies should increase the number of independent plots to better characterise within‐forest‐type variability and enhance generalisability.

While structural metrics are essential for characterising forest ecosystems (LaRue et al. 2023), a more holistic assessment would also integrate vascular plant composition, soil properties and climatic conditions. Since these factors are not included in the SHI assessment, the index provides a valuable yet partial overview of forest structural complexity rather than a comprehensive account of ecosystem functioning.

The considerable residual variation—81.2% in the CCA and 32.4% in the VP analysis—underscores the need for future investigations to explicitly account for additional environmental covariates, particularly disturbance history, edaphic properties and stand‐level microclimate, all of which were unavailable in the present dataset (Thom and Seidl 2016).

Advanced tools—including dendrochronology, soil analysis, satellite‐derived canopy temperature, microclimate loggers, and LiDAR or terrestrial laser scanning—could help capture key ecological processes that extend beyond conventional structural inventories. Integrating these data streams within long‐term monitoring frameworks could help clarify ecological pathways, improve predictive power and strengthen conservation strategies under changing climatic conditions. Overall, long‐term ecological monitoring remains valuable for understanding how forest structure and floristic composition co‐develop under a changing climate. Embedding the structural‐topographic‐floristic framework tested in the Southern Apennines within broader monitoring networks—combining remote sensing, dendrochronological records and in situ microclimate measurements—could refine conservation strategies and adaptive management while allowing its transferability to be evaluated across other ecological regions.

## 6 Conclusion

Our findings indicate that old‐growth forest complexity in the Southern Apennines should be interpreted through the combined consideration of structural, topographic, historical and floristic dimensions. Topography defines part of the environmental and accessibility context (Castellaneta et al. 2023), while old‐growth structural attributes—including large trees, multilayered canopies and deadwood diversity—create microclimatic (Zellweger et al. 2019) and habitat conditions that are closely associated with vascular plant composition.

Across the complete sampled gradient, structural attributes explained more unique floristic variation than topography alone. The SHI provides a useful summary of stand complexity, but its interpretation should remain integrated with topographic context and floristic information.

The mixed‐effects analysis qualified rather than overturned this conclusion. Aspect showed a significant joint association with SHI after accounting for Site, whereas elevation and slope were not significant at the subplot level. Given the hierarchical sampling design, the primary variation partitioning should be interpreted as describing associations across the full Southern Apennine site gradient rather than independent causal effects at the subplot scale.

Conservation priorities should favour stands with abundant large trees (Lindenmayer 2017), diverse deadwood decay stages and multilayered canopies, while recognising that rugged terrain may have indirectly protected these attributes by limiting historical extraction. Where strict protection is not feasible, management should retain old‐growth legacies and promote structural diversification (Mikoláš et al. 2023) without assuming that physical variables alone determine habitat quality. Integration of structural inventories with floristic surveys, land‐use history, soil information, remote sensing, dendrochronology, and microclimate monitoring can strengthen long‐term conservation planning.

These findings support a multidimensional interpretation of old‐growthness that is directly applicable to the studied Southern Apennine forests and generates testable hypotheses for other regions (Tinya et al. 2025). Broader transfer should be validated rather than assumed, because forest type, bioclimate, disturbance history (Thom and Seidl 2016) and socio‐economic context can modify both structural expression and biodiversity responses.

## Author Contributions

**Danilo Travascia:** conceptualization (equal), formal analysis (equal), investigation (equal), methodology (equal), writing – original draft (equal), writing – review and editing (equal). **Gianluca Piovesan:** conceptualization (equal), supervision (equal), writing – review and editing (equal). **Nicodemo Passalacqua:** conceptualization (equal), formal analysis (equal), investigation (equal), methodology (equal), writing – review and editing (equal). **Marco Borghetti:** writing – review and editing (equal). **Liliana Bernardo:** investigation (equal), writing – review and editing (equal). **Sabina Burrascano:** conceptualization (equal), writing – review and editing (equal). **Michele Colangelo:** writing – review and editing (equal). **Costanza Fiorentino:** formal analysis (equal), writing – review and editing (equal). **Domenico Gargano:** investigation (equal), writing – review and editing (equal). **Antonio Lapolla:** investigation (equal). **Vittoria Marchianò:** data curation (equal), investigation (equal). **Anna Rita Rivelli:** investigation (equal), writing – review and editing (equal). **Aldo Schettino:** data curation (equal), investigation (equal). **Francesco Ripullone:** conceptualization (equal), funding acquisition (equal), supervision (equal), writing – review and editing (equal).

## Funding

This research was funded by the Italian Ministry of Environment and Energy Security and the Pollino National Park, within the project ‘Costituzione della rete dei boschi vetusti dei Parchi Nazionali dell'Appennino Meridionale’. The Next Generation EU provided additional support—PNRR Tech4You Project (ECS00000009, CUP C43C22000400006), University of Basilicata.

## Conflicts of Interest

The authors declare no conflicts of interest.

## Supporting information


**Figure S1:** Principal component analysis of forest structural attributes (Dim 1 vs. Dim 2). PCA biplot illustrates the relationships between forest structural attributes, with the first two dimensions, Dim 1 and Dim 2, explaining 44.2% of the total variance. The arrows represent the contribution and direction of each variable to the ordering, with colour intensity showing the relative contribution to the principal components. Variables such as dbh50.sq., dbh40.log and BA_living_tree show strong positive loadings on Dim 1, while dbh_max and Dom_height.sq. are associated more with Dim 2, illustrating gradients in forest structural complexity.
**Figure S2:** Principal component analysis of forest structural attributes (Dim 1 vs. Dim 3). The biplot shows interrelationships among forest structural variables, where Dimension 1 (23.2%) and Dimension 3 (18.7%) together explain 41.9% of the total variance. Variables such as BA_living_tree and dbh40.log load mainly on positive Dim 1, reflecting the tree‐size and basal‐area gradient. In contrast, N_decay_classes and DLR.tu show marked positive loadings towards Dim 3, while DLT.log also contributes to this axis, distinguishing the third component of structural variation.
**Figure S3:** Diagnostic plots for the linear mixed‐effects model fitted to Tukey‐transformed Structural Heterogeneity Index (SHI), with standardised elevation, slope, aspect cosine and aspect sine as fixed effects and Site as a random intercept. The panels evaluate residual patterns and distributional assumptions; the fitted model was non‐singular.
**Figure S4:** Correlations between vascular plant taxon occurrences and the first three PCoA axes. Taxa shown are those retained for interpretation according to the |*r*| > 0.6 criterion. Red indicates positive associations and blue indicates negative associations.
**Figure S5:** Relationship between the Structural Heterogeneity Index (SHI) and PCoA Axis 1 based on the retained primary ordination (142 subplots). Points are coloured by OGF site. Spearman's rank correlation showed no significant association (*ρ* = −0.051, *p* = 0.5495).
**Figure S6:** Relationship between the Structural Heterogeneity Index (SHI) and PCoA Axis 2 based on the retained primary ordination (142 subplots). Points are coloured by OGF site. Spearman's rank correlation showed a positive association (*ρ* = 0.333, *p* = 5.28 × 10⁻⁵).
**Figure S7:** Relationship between the Structural Heterogeneity Index (SHI) and PCoA Axis 3 based on the retained primary ordination (142 subplots). Points are coloured by OGF site. Spearman's rank correlation showed a weaker positive association (*ρ* = 0.185, *p* = 0.0276).
**Figure S8:** Partitioning of vascular plant compositional variation across the complete sampled gradient. Unique structural attributes explained 23.5%, unique topography 12.8%, their shared fraction 31.3% and residual variation 32.4%.
**Figure S9:** Incidence‐based rarefaction and extrapolation of species richness for the pooled unfiltered floristic matrix. The solid line represents rarefaction, the dashed line extrapolation, the point the observed richness at 142 sampling units and the shaded area the 95% confidence interval.
**Figure S10:** Incidence‐based rarefaction and extrapolation by site for the unfiltered floristic matrix. Solid lines represent rarefaction, dashed lines extrapolation, points observed richness and shaded areas 95% confidence intervals. Unequal curve lengths reflect the number of analytical subplots available at each site.
**Figure S11:** Sensitivity of the flora PCoA to alternative taxon incidence thresholds. The ordination outcomes obtained using thresholds ranging from one to five occurrences were compared with the primary analysis, which considered only taxa recorded in at least four subplots. Procrustes correlations quantify the similarity in the three‐dimensional PCoA configuration, while Pearson and Spearman quantify the agreement between pairwise Jaccard dissimilarities. All three metrics were close to 1 across all thresholds, indicating that the primary floristic ordination structure was highly stable with respect to the taxon‐frequency filtering criterion.
**Table S1:** Characteristics of the nine surveyed candidate OGF stands: site abbreviation, forest type, SCI code, coordinates, area, elevation, living‐tree volume (LTV), maximum age, maximum height, maximum diameter at breast height and principal tree species.
**Table S2:** List of structural attributes used in this study at the subplot scale.
**Table S3:** Heterogeneity classes proposed by Sabatini et al. (2015). These classes capture the key structural attributes affecting forest and ecosystem functions. Each class represents a distinct source of heterogeneity, such as vertical stratification, tree species composition, deadwood abundance and tree size distributions, features which contribute to complex and resilient forest ecosystems.
**Table S4:** Partitioning of total CCA inertia into constrained and unconstrained components. The topographic predictor set accounted for 0.09791 of the total inertia (18.79%), whereas 0.42325 (81.21%) remained unconstrained.
**Table S5:** Results from the CCA permutation test. The model comprised 4 degrees of freedom (df = 4) and showed a chi‐squared value of 0.09791 and a high *F*‐ratio (*F* = 7.9809), both of which indicated a significant contribution of the constrained variables. Furthermore, its significant *p*‐value (Pr(>*F*) = 0.001) indicated a significant overall association between the topographic predictor set and forest structural variation.
**Table S6:** Permutation test for the RDA model, including forest structural attributes and topographical variables.
**Table S7:** Linear mixed‐effects model for Tukey‐transformed Structural Heterogeneity Index (SHI). Predictors were standardised, and Site was considered as a random intercept. The joint aspect test removed both cosine and sine components from the full model. Site variance = 99,552; residual variance = 392,214; ICC = 0.202; the model was non‐singular. Raw‐SHI sensitivity tests confirmed the joint aspect effect (*p* = 0.0094) and non‐significant elevation and slope effects.
**Table S8:** Sensitivity of the PCoA to the minimum incidence‐frequency threshold. Procrustes correlations and Pearson/Spearman correlations among Jaccard distance matrices are reported with respect to the primary threshold (four occurrences).
**Table S9:** Observed incidence‐based richness, Chao2 estimates, 95% confidence intervals and sample coverage for the unfiltered floristic matrix. Site‐level uncertainty is wider where fewer analytical subplots were available.

## Data Availability

The data underlying this study are archived in Zenodo as Travascia et al. (2026), *Comprehensive dataset of Southern Apennine old‐growth forests: structure, topography and vascular plant species* (Version 2.0), available at https://doi.org/10.5281/zenodo.21793314. The dataset is currently available upon request and will be made fully public once the research has been published, according to the criteria set by the data owner, the Pollino National Park. All R scripts and reproducible analytical workflows are openly available in Zenodo as Travascia (2026), *Structure, Topography and Flora in Southern Apennine Old‐Growth Forests: An R Workflow* (Version 2.0), available at https://doi.org/10.5281/zenodo.21795886.

## References

[ece374230-bib-0001] Badalamenti, E. , T. La Mantia , G. La Mantia , A. Cairone , and D. S. La Mela Veca . 2017. “Living and Dead Aboveground Biomass in Mediterranean Forests: Evidence of Old‐Growth Traits in a *Quercus pubescens* Willd. S.L. Stand.” Forests 8, no. 6: 187. 10.3390/f8060187.

[ece374230-bib-0002] Baliva, M. , J. Palli , F. Perri , F. Iovino , G. Luzzi , and G. Piovesan . 2024. “The Return of Tall Forests: Reconstructing the Canopy Resilience of an Extensively Harvested Primary Forest in Mediterranean Mountains.” Science of the Total Environment 953: 175806. 10.1016/j.scitotenv.2024.175806. 39197759

[ece374230-bib-0003] Balloffet, N. , and R. K. Dumroese . 2022. “The National Reforestation Strategy and the REPLANT Act: Growing and Nurturing Resilient Forests.” in T. B. Jain and T. M. Schuler, compilers. Foundational Concepts in Silviculture with Emphasis on Reforestation and Early Stand Improvement—2022 National Silviculture Workshop. Proceedings RMRS‐P‐80. U.S. Forest Service, Rocky Mountain Research Station, Fort Collins, Colorado, USA. https://www.usda.gov/sites/default/files/documents/reforestation‐strategy.pdf.

[ece374230-bib-0004] Barnett, K. , G. H. Aplet , and R. T. Belote . 2023. “Classifying, Inventorying, and Mapping Mature and Old‐Growth Forests in the United States.” Frontiers in Forests and Global Change 5: 1070372. 10.3389/ffgc.2022.1070372.

[ece374230-bib-0005] Barredo, J. I. , C. Brailescu , A. Teller , F. M. Sabatini , A. Mauri , and K. Janoušková . 2021. Mapping and Assessment of Primary and Old‐Growth Forests in Europe. Publications Office of the European Union. 10.2760/13239.

[ece374230-bib-0006] Bauhus, J. , K. Puettmann , and C. Messier . 2009. “Silviculture for Old‐Growth Attributes.” Forest Ecology and Management 258, no. 4: 525–537. 10.1016/j.foreco.2009.01.053.

[ece374230-bib-0007] Blasi, C. , M. Marchetti , U. Chiavetta , et al. 2010. “Multi‐Taxon and Forest Structure Sampling for Identification of Indicators and Monitoring of Old‐Growth Forest.” Plant Biosystems 144, no. 1: 160–170. 10.1080/11263500903560538.

[ece374230-bib-0008] Blasi, C. , and L. Michetti . 2005. “Biodiversity and Climate.” In Biodiversity in Italy: Contribution to the National Biodiversity Strategy, 57–66. Palombi Editori. https://www.mase.gov.it/sites/default/files/archivio/biblioteca/protezione_natura/ecoregioni_italia_eng.pdf.

[ece374230-bib-0009] Borghi, C. , S. Francini , R. E. McRoberts , et al. 2024. “Country‐Wide Assessment of Biodiversity, Naturalness, and Old‐Growth Status Using National Forest Inventory Data.” European Journal of Forest Research 143, no. 1: 271–303. 10.1007/s10342-023-01620-6.

[ece374230-bib-0010] Bruening, J. M. , R. O. Dubayah , N. Pederson , B. Poulter , and L. Calle . 2024. “Definition Criteria Determine the Success of Old‐Growth Mapping.” Ecological Indicators 159: 111709. 10.1016/j.ecolind.2024.111709.

[ece374230-bib-0011] Brumelis, G. , B. G. Jonsson , J. Kouki , T. Kuuluvainen , and E. Shorohova . 2011. “Forest Naturalness in Northern Europe: Perspectives on Processes, Structures, and Species Diversity.” Silva Fennica 45, no. 5: 446. 10.14214/sf.446.

[ece374230-bib-0012] Burrascano, S. , W. S. Keeton , F. M. Sabatini , and C. Blasi . 2013. “Commonality and Variability in the Structural Attributes of Moist Temperate Old‐Growth Forests: A Global Review.” Forest Ecology and Management 291: 458–479. 10.1016/j.foreco.2012.11.020.

[ece374230-bib-0013] Castellaneta, M. , A. Schettino , D. Travascia , et al. 2023. “I boschi vetusti nel Parco del Pollino: situazione attuale e prospettive future.” Forest 20: 22–29. 10.3832/efor4303-020.

[ece374230-bib-0014] Chahouki, M. A. Z. 2011. “Multivariate analysis techniques in environmental science.” In Earth and Environmental Sciences, edited by I. A. Dar and M. A. Dar , 539–564. IntechOpen. 10.5772/26516.

[ece374230-bib-0015] Chao, A. 1987. “Estimating the Population Size for Capture‐Recapture Data With Unequal Catchability.” Biometrics 43, no. 4: 783–791. 10.2307/2531532. 3427163

[ece374230-bib-0016] Chećko, E. , B. Jaroszewicz , K. Olejniczak , and A. J. Kwiatkowska‐Falińska . 2015. “The Importance of Coarse Woody Debris for Vascular Plants in Temperate Mixed Deciduous Forests.” Canadian Journal of Forest Research 45, no. 9: 1154–1163. 10.1139/cjfr-2014-0473.

[ece374230-bib-0017] Chirici, G. , S. Winter , and R. E. McRoberts . 2011. National Forest Inventories: Contributions to Forest Biodiversity Assessments. Springer. 10.1007/978-94-007-0482-4.

[ece374230-bib-0018] Chytrý, M. , M. Řezníčková , P. Novotný , et al. 2024. “FloraVeg.EU: An Online Database of European Vegetation, Habitats, and Flora.” Applied Vegetation Science 27: e12798. 10.1111/avsc.12798.

[ece374230-bib-0019] Colangelo, M. , J. J. Camarero , A. Gazol , et al. 2021. “Mediterranean Old‐Growth Forests Exhibit Resistance to Climate Warming.” Science of the Total Environment 801: 149684. 10.1016/j.scitotenv.2021.149684. 34467901

[ece374230-bib-0020] Compagnucci, F. , and F. Mazzoni . 2002. “Il territorio dei Parchi Nazionali Italiani.” In Quaderni di Ricerca, vol. 172. Università degli Studi di Ancona, Dipartimento di Economia. https://ideas.repec.org/p/anc/wpaper/172.html.

[ece374230-bib-0021] Concha, V. C. , J. Caviedes , F. J. Novoa , T. A. Altamirano , and J. T. Ibarra . 2023. “Structural Complexity Is a Better Predictor Than Single Habitat Attributes of Understory Bird Densities in Andean Temperate Forests.” Ornithological Applications 125, no. 4: duad035. 10.1093/ornithapp/duad035.

[ece374230-bib-0022] De Frenne, P. , J. Lenoir , M. Luoto , et al. 2021. “Forest Microclimates and Climate Change: Importance, Drivers and Future Research Agenda.” Global Change Biology 27, no. 11: 2279–2297. 10.1111/gcb.15569. 33725415

[ece374230-bib-0023] Di Pietro, R. 2009. “Observations on the Beech Woodlands of the Apennines (Peninsular Italy): An Intricate Biogeographical and Syntaxonomical Issue.” Mediterranean Botany 30: 89. https://www.researchgate.net/publication/272805949.

[ece374230-bib-0024] Dillenberger, M. S. , and J. W. Kadereit . 2013. “The Phylogeny of the European High Mountain Genus *Adenostyles* (Asteraceae–Senecioneae) Reveals That Edaphic Shifts Coincide With Dispersal Events.” American Journal of Botany 100, no. 6: 1171–1183. 10.3732/ajb.1300060. 23709635

[ece374230-bib-0025] Fantini, S. , M. Fois , P. Casula , G. Fenu , G. Calvia , and G. Bacchetta . 2020. “Structural Heterogeneity and Old‐Growthness: A First Regional‐Scale Assessment of Sardinian Forests.” Annals of Forest Research 63, no. 2: 103–120. 10.15287/afr.2020.1968.

[ece374230-bib-0026] Filibeck, G. , J. Adams , M. Brunetti , et al. 2015. “Tree‐Ring Ecological Signal Is Consistent With Floristic Composition and Plant Indicator Values in Mediterranean *Fagus sylvatica* Forests.” Journal of Ecology 103, no. 6: 1580–1593. 10.1111/1365-2745.12478.

[ece374230-bib-0027] Forest Europe . 2020. “State of Europe's Forests 2020.” https://foresteurope.org/wp‐content/uploads/2016/08/SoEF_2020.pdf.

[ece374230-bib-0028] Franklin, J. F. , T. A. Spies , R. Van Pelt , et al. 2002. “Disturbances and Structural Development of Natural Forest Ecosystems With Silvicultural Implications, Using Douglas‐Fir Forests as an Example.” Forest Ecology and Management 155: 399–423. 10.1016/S0378-1127(01)00575-8.

[ece374230-bib-0029] Gilhen‐Baker, M. , V. Roviello , D. Beresford‐Kroeger , and G. N. Roviello . 2022. “Old‐Growth Forests and Large Old Trees as Critical Organisms Connecting Ecosystems and Human Health: A Review.” Environmental Chemistry Letters 20, no. 2: 1529–1538. 10.1007/s10311-021-01372-y. 35002589 PMC8728480

[ece374230-bib-0030] Giorgini, D. , P. Giordani , G. Casazza , V. Amici , M. G. Mariotti , and A. Chiarucci . 2015. “Woody Species Diversity as Predictor of Vascular Plant Species Diversity in Forest Ecosystems.” Forest Ecology and Management 345: 50–55. 10.1016/j.foreco.2015.02.016.

[ece374230-bib-0031] Goslee, S. C. , and D. L. Urban . 2007. “The Ecodist Package for Dissimilarity‐Based Analysis of Ecological Data.” Journal of Statistical Software 22: 1–19. 10.18637/jss.v022.i07.

[ece374230-bib-0032] Govedar, Z. , S. Vojniković , and N. Velkovski . 2024. “Old‐Growth Forests of Southeast Europe and Their Relevance for Forest Management.” Frontiers in Forests and Global Change 7: 1447181. 10.3389/ffgc.2024.1447181.

[ece374230-bib-0033] Graf, M. , S. Seibold , M. M. Gossner , et al. 2022. “Coverage‐Based Diversity Estimates of Facultative Saproxylic Species Highlight the Importance of Deadwood for Biodiversity.” Forest Ecology and Management 517: 120275. 10.1016/j.foreco.2022.120275.

[ece374230-bib-0034] Gray, A. N. , K. Pelz , G. D. Hayward , et al. 2023. “Perspectives: The Wicked Problem of Defining and Inventorying Mature and Old‐Growth Forests.” Forest Ecology and Management 546: 121350. 10.1016/j.foreco.2023.121350.

[ece374230-bib-0035] Hsieh, T. C. , K. H. Ma , and A. Chao . 2016. “iNEXT: An R Package for Rarefaction and Extrapolation of Species Diversity (Hill Numbers).” Methods in Ecology and Evolution 7: 1451–1456. 10.1111/2041-210X.12613.

[ece374230-bib-0036] Hunter, M. L. 1990. Wildlife, Forests and Forestry: Principles for Managing Forests for Biological Diversity. Prentice Hall. https://nnrg.org/wp‐content/uploads/2015/03/seymourchapter.pdf.

[ece374230-bib-0037] Hurlbert, S. H. 1984. “Pseudoreplication and the Design of Ecological Field Experiments.” Ecological Monographs 54, no. 2: 187–211. 10.2307/1942661.

[ece374230-bib-0038] Jackson, T. D. , F. J. Fischer , G. Vincent , et al. 2024. “Tall Bornean Forests Experience Higher Canopy Disturbance Rates Than Those in the Eastern Amazon or Guiana Shield.” Global Change Biology 30, no. 9: e17493. 10.1111/gcb.17493. 39239723

[ece374230-bib-0039] Jucker, T. , B. Bongalov , D. F. Burslem , et al. 2018. “Topography Shapes the Structure, Composition and Function of Tropical Forest Landscapes.” Ecology Letters 21, no. 7: 989–1000. 10.1111/ele.12964. 29659115 PMC6849614

[ece374230-bib-0040] Kassambara, A. , and F. Mundt . 2017. “Factoextra: Extract and Visualize the Results of Multivariate Data Analyses.” R package version 1.0–7. https://rpkgs.datanovia.com/factoextra/.

[ece374230-bib-0041] Keenan, R. J. , and S. M. Read . 2012. “Assessment and Management of Old‐Growth Forests in South‐Eastern Australia.” Plant Biosystems 146, no. 1: 214–222. 10.1080/11263504.2011.650726.

[ece374230-bib-0042] LaRue, E. A. , J. A. Knott , G. M. Domke , et al. 2023. “Structural Diversity as a Reliable and Novel Predictor for Ecosystem Productivity.” Frontiers in Ecology and the Environment 21, no. 1: 33–39. 10.1002/fee.2586.

[ece374230-bib-0043] Lê, S. , J. Josse , and F. Husson . 2008. “FactoMineR: An R Package for Multivariate Analysis.” Journal of Statistical Software 25, no. 1: 1–18. 10.18637/jss.v025.i01.

[ece374230-bib-0044] Lindenmayer, D. , and E. Bowd . 2022. “Critical Ecological Roles, Structural Attributes and Conservation of Old‐Growth Forest: Lessons From a Case Study of Australian Mountain Ash Forests.” Frontiers in Forests and Global Change 5: 878570. 10.3389/ffgc.2022.878570.

[ece374230-bib-0045] Lindenmayer, D. B. 2017. “Conserving Large Old Trees as Small Natural Features.” Biological Conservation 211: 51–59. 10.1016/j.biocon.2016.11.012.

[ece374230-bib-0046] Lutz, J. A. , T. J. Furniss , D. J. Johnson , et al. 2018. “Global Importance of Large‐Diameter Trees.” Global Ecology and Biogeography 27, no. 7: 849–864. 10.1111/geb.12747.

[ece374230-bib-0047] Luyssaert, S. , E. D. Schulze , A. Börner , et al. 2008. “Old‐Growth Forests as Global Carbon Sinks.” Nature 455, no. 7210: 213–215. 10.1038/nature07276. 18784722

[ece374230-bib-0048] Matuszkiewicz, J. M. , A. N. Affek , P. Zaniewski , E. Kołaczkowska , and E. N. Kołaczkowska . 2024. “Early Response of Understory Vegetation to the Mass Dieback of Norway Spruce in the European Lowland Temperate Forest.” Forest Ecosystems 11: 100177. 10.1016/j.fecs.2024.100177.

[ece374230-bib-0049] McCune, B. , and J. B. Grace . 2002. Analysis of Ecological Communities. MjM Software Design.

[ece374230-bib-0050] McElhinny, C. , P. Gibbons , and C. Brack . 2006. “An Objective and Quantitative Methodology for Constructing an Index of Stand Structural Complexity.” Forest Ecology and Management 235, no. 1–3: 54–71. 10.1016/j.foreco.2006.07.024.

[ece374230-bib-0051] McNichol, B. H. , R. Wang , A. Hefner , C. Helzer , S. M. McMahon , and S. E. Russo . 2024. “Topography‐Driven Microclimate Gradients Shape Forest Structure, Diversity, and Composition in a Temperate Refugial Forest.” Plant‐Environment Interactions 5, no. 3: e10153. 10.1002/pei3.10153. 38863691 PMC11166229

[ece374230-bib-0052] Mikoláš, M. , G. Piovesan , A. Ahlström , et al. 2023. “Protect Old‐Growth Forests in Europe Now.” Science 380, no. 6644: 466. 10.1126/science.adh2303. 37141361

[ece374230-bib-0053] Müller, J. , and R. Bütler . 2010. “A Review of Habitat Thresholds for Dead Wood: A Baseline for Management Recommendations in European Forests.” European Journal of Forest Research 129, no. 6: 981–992. 10.1007/s10342-010-0400-5.

[ece374230-bib-0054] O'Brien, L. , A. Schuck , C. Fraccaroli , E. Pötzelsberger , G. Winkel , and M. Lindner . 2021. “Protecting Old‐Growth Forests in Europe: A Review of Scientific Evidence to Inform Policy Implementation.” European Forest Institute. 10.36333/rs1.

[ece374230-bib-0055] Oksanen, J. , G. Simpson , F. Blanchet , et al. 2024. “vegan: Community Ecology Package.” R package version 2.7–0. https://github.com/vegandevs/vegan.

[ece374230-bib-0056] Oliver, C. D. , and B. C. Larson . 1996. Forest Stand Dynamics. Yale School of the Environment. https://elischolar.library.yale.edu/fes_pubs/1.

[ece374230-bib-0057] Ontl, T. A. , M. K. Janowiak , C. W. Swanston , et al. 2020. “Forest Management for Carbon Sequestration and Climate Adaptation.” Journal of Forestry 118, no. 1: 86–101. 10.1093/jofore/fvz062.

[ece374230-bib-0058] Paradis, E. , and K. Schliep . 2019. “Ape 5.0: An Environment for Modern Phylogenetics and Evolutionary Analyses in R.” Bioinformatics 35: 526–528. 10.1093/bioinformatics/bty633. 30016406

[ece374230-bib-0059] Pasques, O. , and S. Munné‐Bosch . 2024. “Ancient Trees Are Essential Elements for High‐Mountain Forest Conservation: Linking the Longevity of Trees to Their Ecological Function.” Proceedings of the National Academy of Sciences of the United States of America 121, no. 7: e2317866121. 10.1073/pnas.2317866121. 38315840 PMC10873607

[ece374230-bib-0060] Pereira, H. M. , I. M. D. Rosa , I. S. Martins , et al. 2020. “Global Trends in Biodiversity and Ecosystem Services From 1900 to 2050.” *bioRxiv*. 10.1101/2020.04.14.031716. 38662818

[ece374230-bib-0061] Peruzzi, L. , G. Domina , F. Bartolucci , et al. 2015. “An Inventory of the Names of Vascular Plants Endemic to Italy, Their Loci Classici and Types.” Phytotaxa 196, no. 1: 1–217. 10.11646/phytotaxa.196.1.1.

[ece374230-bib-0062] Pickering, M. , A. Elia , M. Girardello , et al. 2024. “Assessing the Relationship Between Forest Structural Diversity and Resilience in a Warming Climate.” EGUsphere EGU24–16017. 10.5194/egusphere-egu24-16017.

[ece374230-bib-0063] Piovesan, G. , M. Baliva , L. Calcagnile , et al. 2020. “Radiocarbon Dating of Aspromonte Sessile Oaks Reveals the Oldest Dated Temperate Flowering Tree in the World.” Ecology 101, no. 12: e03179. 10.1002/ecy.3179. 32860441

[ece374230-bib-0064] Piovesan, G. , F. Biondi , M. Baliva , et al. 2018. “The Oldest Dated Tree of Europe Lives in the Wild Pollino Massif.” Ecology 99, no. 7: 1682–1684. 10.1002/ecy.2231. 29768656

[ece374230-bib-0065] Piovesan, G. , F. Biondi , M. Baliva , et al. 2019. “Lessons From the Wild.” Ecology 100, no. 9: e02737. 10.1002/ecy.2737. 31135954

[ece374230-bib-0066] Piovesan, G. , F. Biondi , M. Baliva , et al. 2019. “Tree Growth Patterns Associated With Extreme Longevity: Implications for the Ecology and Conservation of Primeval Trees in Mediterranean Mountains.” Anthropocene 26: 100199. 10.1016/j.ancene.2019.100199.

[ece374230-bib-0067] Pörtner, H. O. , R. J. Scholes , J. Agard , et al. 2021. IPBES–IPCC Co‐Sponsored Workshop Report on Biodiversity and Climate Change. IPBES Secretariat. https://library.wur.nl/WebQuery/wurpubs/592922.

[ece374230-bib-0068] R Core Team . 2023. “R: A Language and Environment for Statistical Computing.” R Foundation for Statistical Computing, Vienna, Austria. https://www.R‐project.org/.

[ece374230-bib-0069] Ramette, A. 2007. “Multivariate Analyses in Microbial Ecology.” FEMS Microbiology Ecology 62, no. 2: 142–160. 10.1111/j.1574-6941.2007.00375.x. 17892477 PMC2121141

[ece374230-bib-0070] Ripullone, F. , T. Gentilesca , M. Lauteri , et al. 2016. “Apical Dominance Ratio as an Indicator of the Growth Conditions Favouring *Abies alba* Natural Regeneration Under Mediterranean Environment.” European Journal of Forest Research 135: 377–387. 10.1007/s10342-016-0941-3.

[ece374230-bib-0071] Sabatini, F. M. , H. Bluhm , Z. Kun , et al. 2020. “European Primary Forest Database (EPFD) v2.0.” *bioRxiv*. 10.1101/2020.10.30.362434.

[ece374230-bib-0072] Sabatini, F. M. , S. Burrascano , F. Lombardi , G. Chirici , and C. Blasi . 2015. “An Index of Structural Complexity for Apennine Beech Forests.” iForest 8: 314–323. 10.3832/ifor1160-008.

[ece374230-bib-0073] Selvi, F. , M. Cabrucci , G. Dadà , and E. Carrari . 2026. “Investigating Old‐Growth Forests in Tuscany (Italy): Structural Heterogeneity and Plant Diversity Across Forest Types and Novel Candidate Sites for the National Network.” Land 15, no. 4: 640. 10.3390/land15040640.

[ece374230-bib-0074] Slezák, M. , and I. Axmanová . 2016. “Patterns of Plant Species Richness and Composition in Deciduous Oak Forests in Relation to Environmental Drivers.” Community Ecology 17, no. 1: 61–70. 10.1556/168.2016.17.1.8.

[ece374230-bib-0075] Spies, T. A. , and J. F. Franklin . 1991. “The Structure of Natural Young, Mature, and Old‐Growth Douglas‐Fir Forests in Oregon and Washington.” In Wildlife and Vegetation of Unmanaged Douglas‐Fir Forests, 91–109. Pacific Northwest Research Station, USDA Forest Service. https://andrewsforest.oregonstate.edu/publications/1244.

[ece374230-bib-0076] Storch, F. , S. Boch , M. M. Gossner , et al. 2023. “Linking Structure and Species Richness to Support Forest Biodiversity Monitoring at Large Scales.” Annals of Forest Science 80, no. 1: 3. 10.1186/s13595-022-01169-1.

[ece374230-bib-0077] Tanioka, K. , and H. Yadohisa . 2012. “Effect of Data Standardization on the Result of k‐Means Clustering.” In Challenges at the Interface of Data Analysis, Computer Science, and Optimization, edited by W. Gaul , A. Geyer‐Schulz , L. Schmidt‐Thieme , and J. Kunze , 59–67. Springer. 10.1007/978-3-642-24466-7_7.

[ece374230-bib-0078] Thom, D. , and R. Seidl . 2016. “Natural Disturbance Impacts on Ecosystem Services and Biodiversity in Temperate and Boreal Forests.” Biological Reviews 91, no. 3: 760–781. 10.1111/brv.12193. 26010526 PMC4898621

[ece374230-bib-0079] Tilman, D. 1994. “Competition and Biodiversity in Spatially Structured Habitats.” Ecology 75, no. 1: 2–16. 10.2307/1939377.

[ece374230-bib-0080] Tinya, F. , P. Csépányi , C. V. Horváth , B. Kovács , C. Németh , and P. Ódor . 2025. “Fine‐Scale Interventions Can Reinforce the Forest Character of the Understory Vegetation–the Effects of Different Artificial Gaps in an Oak‐Dominated Forest.” Forest Ecology and Management 578: 122471. 10.1016/j.foreco.2024.122471.

[ece374230-bib-0081] Todaro, L. , L. Andreu , C. M. D'Alessandro , E. Gutiérrez , P. Cherubini , and A. Saracino . 2007. “Response of *Pinus leucodermis* to Climate and Anthropogenic Activity in the National Park of Pollino (Basilicata, Southern Italy).” Biological Conservation 137, no. 4: 507–519. 10.1016/j.biocon.2007.03.010.

[ece374230-bib-0082] van Galen, L. G. , G. J. Jordan , R. A. Musk , N. J. Beeton , T. J. Wardlaw , and S. C. Baker . 2018. “Quantifying Floristic and Structural Forest Maturity: An Attribute‐Based Method for Wet Eucalypt Forests.” Journal of Applied Ecology 55, no. 4: 1668–1681. 10.1111/1365-2664.13133.

[ece374230-bib-0083] Whitman, A. , and J. M. Hagan . 2007. “An Index to Identify Late‐Successional Forest in Temperate and Boreal Zones.” Forest Ecology and Management 246, no. 2–3: 144–154. 10.1016/j.foreco.2007.03.004.

[ece374230-bib-0084] Zellweger, F. , D. Coomes , J. Lenoir , et al. 2019. “Seasonal Drivers of Understorey Temperature Buffering in Temperate Deciduous Forests Across Europe.” Global Ecology and Biogeography 28, no. 12: 1774–1786. 10.1111/geb.12991. 31866760 PMC6900070

[ece374230-bib-0085] Ziaco, E. , A. Di Filippo , A. Alessandrini , M. Baliva , E. D'Andrea , and G. Piovesan . 2012. “Old‐Growth Attributes in a Network of Apennines (Italy) Beech Forests: Disentangling the Role of Past Human Interferences and Biogeoclimate.” Plant Biosystems 146, no. 1: 153–166. 10.1080/11263504.2011.650729.

