# Supplementary material for: Structural Attributes Outweigh Topographical Effects in Determining Vascular Plant Diversity Within Southern Apennine Old‐Growth Forests
Source: Ecol Evol. 2026 Sep 1;16(9):e74230. doi: 10.1002/ece3.74230 (PMC13533614; doi:10.1002/ece3.74230)
Supplement: Supplementary file 1 [file ECE3-16-e74230-s001.docx]

**Figure S1.** Principal component analysis of forest structural attributes (Dim 1 vs. Dim 2). PCA biplot illustrates the relationships between forest structural attributes, with the first two dimensions, Dim 1 and Dim 2, explaining 44.2% of the total variance. The arrows represent the contribution and direction of each variable to the ordering, with colour intensity showing the relative contribution to the principal components. Variables such as dbh50.sq, dbh40.log, and BA_living_tree show strong positive loadings on Dim 1, while dbh_max and Dom_height.sq are associated more with Dim 2, illustrating gradients in forest structural complexity.

**Figure S2.** Principal component analysis of forest structural attributes (Dim 1 vs. Dim 3). The biplot shows interrelationships among forest structural variables, where Dimension 1 (23.2%) and Dimension 3 (18.7%) together explain 41.9% of the total variance. Variables such as BA_living_tree and dbh40.log load mainly on positive Dim 1, reflecting the tree-size and basal-area gradient. In contrast, N_decay_classes and DLR.tu show marked positive loadings toward Dim 3, while DLT.log also contributes to this axis, distinguishing the third component of structural variation.

**Figure S3.** Diagnostic plots for the linear mixed-effects model fitted to Tukey-transformed SHI, with standardised elevation, slope, aspect cosine, and aspect sine as fixed effects and Site as a random intercept. The panels evaluate residual patterns and distributional assumptions; the fitted model was non-singular.

**Figure S4.** Correlations between vascular plant taxon occurrences and the first three PCoA axes. Taxa shown are those retained for interpretation according to the |r| > 0.6 criterion. Red indicates positive associations and blue indicates negative associations.

**Figure S5.** Relationship between the Structural Heterogeneity Index (SHI) and PCoA Axis 1 based on the retained primary ordination (142 subplots). Points are coloured by OGF site. Spearman’s rank correlation showed no significant association (ρ = -0.051, p = 0.5495).

**Figure S6.** Relationship between the Structural Heterogeneity Index (SHI) and PCoA Axis 2 based on the retained primary ordination (142 subplots). Points are coloured by OGF site. Spearman’s rank correlation showed a positive association (ρ = 0.333, p = 5.28 × 10⁻⁵).

**Figure S7.** Relationship between the Structural Heterogeneity Index (SHI) and PCoA Axis 3 based on the retained primary ordination (142 subplots). Points are coloured by OGF site. Spearman’s rank correlation showed a weaker positive association (ρ = 0.185, p = 0.0276).

**Figure S8.** Partitioning of vascular plant compositional variation across the complete sampled gradient. Unique structural attributes explained 23.5%, unique topography 12.8%, their shared fraction 31.3%, and residual variation 32.4%.

**Figure S9.** Incidence-based rarefaction and extrapolation of species richness for the pooled unfiltered floristic matrix. The solid line represents rarefaction, the dashed line extrapolation, the point the observed richness at 142 sampling units, and the shaded area the 95% confidence interval.

**Figure S10**. Incidence-based rarefaction and extrapolation by site for the unfiltered floristic matrix. Solid lines represent rarefaction, dashed lines extrapolation, points observed richness, and shaded areas 95% confidence intervals. Unequal curve lengths reflect the number of analytical subplots available at each site.

**Figure S11. Sensitivity of the flora PCoA to alternative taxon incidence thresholds**. The ordination outcomes obtained using thresholds ranging from one to five occurrences were compared with the primary analysis, which considered only taxa recorded in at least four subplots. Procrustes correlations quantify the similarity in the three-dimensional PCoA configuration, while Pearson and Spearman quantify the agreement between pairwise Jaccard dissimilarities. All three metrics were close to 1 across all thresholds, indicating that the primary floristic ordination structure was highly stable with respect to the taxon-frequency filtering criterion.

| **Abb.** | **Forest type** | **SCI code** | **Coordinates** | | **Area (ha)** | **Elevation (m)** | **LTV (m^3)** | **Max Age** | **Max height** | **Max diameter** | **Main and sporadic tree species** |
| --- | --- | --- | --- | --- | --- | --- | --- | --- | --- | --- | --- |
|  |  |  | ***Lat*** | ***Long*** |  |  |  |  |  |  |  |
| **BV** | Holm-oak forest | IT9310032 | 39,685 | 15,889 | 20 | 850 | 551 | 140 | 20 | 150 | **Holm oak forest. Dominant**: *Quercus ilex* L. **Sporadic**: *Acer campestre* L.; *Fraxinus ornus* L.; *Crataegus monogyna* Jacq.; *Alnus cordata* L.; and *Quercus pubescens* Willd. |
| **BM** | Mixed forest | IT9210040 | 40,05 | 16,105 | 18 | 850 | 516 | 261 | 50 | 150 | **Mixed forest. Codominant**: *Fagus sylvatica* L. and *Quercus cerris* L. **Sporadic**: *Abies alba* Mill.; *Carpinus betulus* L.; *Alnus glutinosa* L.; and *Ilex aquifolium* L. |
| **CA** | Mixed forest | IT9210070 | 40,124 | 16,048 | 21 | 950 | 349 | 231 | 30 | 160 | **Mixed forest Codominant**: *Fagus sylvatica* L., and *Abies alba* Mill. **Sporadic**: *Alnus cordata* Loisel.; *Acer campestre* L.; *Quercus cerris* L.; *Quercus pubescens* Willd.; and *Ilex aquifolium* L. |
| **AC** | Maple forest | IT9310019 | 39,928 | 16,354 | 72 | 1300 | 478 | 187 | 29 | 110 | **Maple forest. Codominant**: *Acer campestre* L.*; Acer opalus* Mill.*; Acer pseudoplatanus* L. **Sporadic**: *Fagus sylvatica* L.; *Abies alba* Mill.; and *Alnus cordata* Loisel. |
| **TP** | Mixed forest | IT9210075 | 39,957 | 16,216 | 83 | 1400 | 635 | 329 | 36 | 110 | **Codominant:** *Abies alba* Mill.; and *Fagus sylvatica* L. **Sporadic**: *Pyrus communis* L.; and *Quercus cerris* |
| **RO** | Beech forest | 1133ter-045 | 39,906 | 16,094 | 70 | 1750 | 582 | 398 | 28 | 125 | **Pure beech:** *Fagus sylvatica* L |
| **VIG** | Beech forest | IT9210125 | 39,936 | 16,117 | 54 | 1750 | 388 | 428 | 26 | 85 | **Pure**: *Fagus sylvatica* L.; **Sporadic**: *Acer cappadocicum* Gled. subsp*. lobelii* |
| **PO** | Beech forest | 1133qui-082 | 39,896 | 16,167 | 20 | 1950 | 450 | 400 | 21 | 68 | **Pure**: *Fagus sylvatica* L |
| **SC*** | Bosnian Pine | IT9210245 | 39,935 | 16,21 | 100 | 2050 | 53 | 305 | 14 | 160 | **Pure:** [*Pinus heldreichii* Christ*.* subsp*. leucodermis*](https://dryades.units.it/casentinesi/index.php?procedure=taxon_page&id=161&num=2909). |

**Table S1**. Characteristics of the nine surveyed candidate OGF stands: site abbreviation, forest type, SCI code, coordinates, area, elevation, living-tree volume (LTV), maximum age, maximum height, maximum diameter at breast height, and principal tree species.

| **Abbreviation** | **Variable** | **Unit of measure** |
| --- | --- | --- |
| **BA_living_tree** | Basal area living tree | m^2^ |
| **BASnagST** | Basal area of snags and Dead standing trees | m^2^ |
| **Canopy_cover** | Canopy cover | % |
| **Vol_CWD** | Volume Coarse Woody Debris | m^3^ |
| **Density_living_tree** | No. Living trees | No. trees |
| **DensSnagST** | No. Snag & Dead standing trees | No. trees |
| **Vol_necromass** | Volume necromass | m^3^ |
| **Vol_living_trees** | Volume of living trees | m^3^ |
| **N_dbh** | No. Diameter class | No. classes |
| **N_decay_classes** | No. Decay classes | No. classes |
| **Vol_SnagST** | Volume Snag and Dead standing tree | m^3^ |
| **n_dbh_40** | No. Tree with DBH > 40 | No. trees |
| **n_dbh_50** | No. Tree with DBH > 50 | No. trees |
| **n_dbh_70** | No. Tree with DBH > 70 | No. trees |
| **Tree_rich** | No. Tree species | No. tree species |
| **Vol_stump** | Volume stump | m^3^ |
| **Vol_ddt** | Volume of downed dead tree | m^3^ |
| **Dom_height** | Average of the 5 highest trees | m |
| **QMD** | Quadratic mean diameter | cm |
| **H_sd** | Height Standard deviation | m |
| **Gini** | Gini index (basal area) | - |
| **dlR** | Dead-to-live ratio | - |
| **dbh_max** | Max diameter | cm |
| **dbh_min** | Min diameter | cm |

**Table S2**. List of structural attributes used in this study at the subplot scale.

| **Source of Heterogeneity** | **Description** | **Selected Variable** |
| --- | --- | --- |
| **Vertical Heterogeneity** | Stands with a variety of tree heights likely have a mix of ages, leading to high vertical and horizontal variation. This variation influences demographics, resource distribution (light), and understorey development. | H_sd, Dom_height |
| **Tree Compositional Diversity** | A mix of shade-tolerant and shade-intolerant species can create a multi-layered canopy. This diversity can promote herb-layer variety due to differences in light transmission and litter quality between tree species. |  |
| **Uneven-agedness** | In landscapes with frequent small-scale disturbances, an uneven-aged structure might be natural or due to close-to-nature forestry practices. The variation in tree sizes indicates a diversity of niches for various plants and animals. | DLT.log |
| **Density of Large Living Trees** | Large trees store significant carbon and provide habitat for threatened or ecologically important species by offering a variety of niches, i.e., rough bark, hollows, exposed deadwood, sap flows, and dead branches/tops. | dbh40.log,  dbh50.sq, |
| **Growing Stock** | Living above-ground biomass indicates a stand's carbon storage. Older-growth stands, with a higher biomass, more effectively moderate the surface temperature and support a higher percentage of forest specialist herbaceous species. | dbh_max, BA_living_tree |
| **Total Deadwood Volume** | Deadwood is crucial for biodiversity by providing niches for specialised organisms. These organisms often have limited dispersal abilities and rely on the long-term availability of deadwood for survival. Their absence in managed stands can lead to local extinctions. |  |
| **Deadwood Decay Classes** | The lack of deadwood in specific decay stages indicates a disruption in its supply, usually due to recent harvesting or removal. This can affect nutrient cycling and the diversity/abundance of saproxylic organisms. | N_decay_classes |
| **Standing Deadwood, Dead Trees, and Snags** | Standing dead trees and snags offer niches like hollows, cracks, and cavity strings, which are vital for various species of breeding birds, mammals, invertebrates, lichens, and bryophytes. | DLR.tu |

**Table S3**. Heterogeneity classes proposed by Sabatini et al. (2015). These classes capture the key structural attributes affecting forest biodiversity and ecosystem functions. Each class represents a distinct source of heterogeneity, such as vertical stratification, tree species composition, deadwood abundance, and tree size distributions, features which contribute to complex and resilient forest ecosystems.

| **Partitioning of scaled Chi-square** | | |
| --- | --- | --- |
|  | *Inertia* | *Proportion* |
| **Total** | 0.52116 | 1.0000 |
| **Constrained** | 0.09791 | 0.1879 |
| **Unconstrained** | 0.42325 | 0.8121 |

**Table S4**. Partitioning of total CCA inertia into constrained and unconstrained components. The topographic predictor set accounted for 0.09791 of the total inertia (18.79%), whereas 0.42325 (81.21%) remained unconstrained.

| **Permutation test for CCA under reduced model** | | | | |
| --- | --- | --- | --- | --- |
| **Number of permutations: 999** | | | | |
|  | **Df** | **ChiSquare** | **F** | **Pr(>F)** |
| **Model** | 4 | 0.09791 | 7.9809 | 0.001 |
| **Residual** | 138 | 0.42325 |  |  |

**Table S5**. Results from the CCA permutation test. The model comprised 4 degrees of freedom (Df = 4) and showed a Chi-square value of 0.09791 and a high F-ratio (F = 7.9809), both of which indicated a significant contribution of the constrained variables. Furthermore, its significant p-value (Pr(>F) = 0.001) indicated a significant overall association between the topographic predictor set and forest structural variation.

| **Permutation test for RDA under reduced model** | | | | |
| --- | --- | --- | --- | --- |
| **Number of permutations: 999** | | | | |
|  | **Df** | **Variance** | **F** | **Pr(>F)** |
| **Model** | 19 | 0.155842 | 16.512 | 0.001 *** |
| **Residual** | 122 | 0.060603 |  |  |

**Table S6**. Permutation test for the RDA model, including forest structural attributes and topographical variables.

| Effect | Estimate (SE) | t (*df*) | Coefficient p | Partial LRT χ² (*df*), p |
| --- | --- | --- | --- | --- |
| **Elevation** | 174.025 (126.691) | 1.374 (9.189) | 0.2021 | 1.639 (1), p = 0.2005 |
| **Slope** | 24.614 (70.827) | 0.348 (124.278) | 0.7288 | 0.104 (1), p = 0.7469 |
| **Aspect cosine** | -194.555 (62.290) | -3.123 (141.865) | 0.0022 | — |
| **Aspect sine** | -13.093 (81.870) | -0.160 (133.986) | 0.8732 | — |
| **Aspect, joint test** | — | — | — | 10.184 (2), p = 0.0061 |

**Table S7.** Linear mixed-effects model for Tukey-transformed SHI. Predictors were standardised, and Site was considered as a random intercept. The joint aspect test removed both cosine and sine components from the full model. Site variance = 99,552; residual variance = 392,214; ICC = 0.202; the model was non-singular. Raw-SHI sensitivity tests confirmed the joint aspect effect (p = 0.0094) and non-significant elevation and slope effects.

| Frequency | Taxa | Variance  PCoA1 / PCoA2 / PCoA3 (%) | Procrustes r | Jaccard Pearson / Spearman |
| --- | --- | --- | --- | --- |
| 1 | 153 | 14.64 / 12.62 / 8.01 | 0.9992 | 0.9954 / 0.9970 |
| 2 | 134 | 14.72 / 12.72 / 8.07 | 0.9994 | 0.9966 / 0.9978 |
| 3 | 118 | 14.91 / 12.91 / 8.13 | 0.9996 | 0.9980 / 0.9989 |
| 4 (primary) | 100 | 15.22 / 13.40 / 8.37 | 1.0000 | 1.0000 / 1.0000 |
| 5 | 84 | 15.69 / 13.80 / 8.61 | 0.9997 | 0.9971 / 0.9988 |

**Table S8.** Sensitivity of the PCoA to the minimum incidence-frequency threshold. Procrustes correlations and Pearson/Spearman correlations among Jaccard distance matrices are reported with respect to the primary threshold (four occurrences).

| Assemblage | Observed taxa | Chao2 (SE) | 95% CI | Sample coverage |
| --- | --- | --- | --- | --- |
| Pooled | 153 | 170.509 (9.553) | 159.438–200.620 | 0.9865 |
| AC | 55 | 66.520 (8.825) | 58.041–98.636 | 0.9726 |
| BM | 60 | 68.553 (6.009) | 62.472–89.596 | 0.9504 |
| CA | 24 | 56.000 (29.755) | 30.812–17*4.3*23 | 0.8255 |
| RO | 53 | 57.278 (3.642) | 54.007–71.183 | 0.9678 |
| TP | 29 | 29.617 (1.086) | 29.060–35.327 | 0.9864 |
| PO | 29 | 34.625 (6.105) | 29.998–60.700 | 0.9710 |
| BV | 41 | 64.207 (14.613) | 48.477–113.031 | 0.8261 |
| VIG | 32 | 35.360 (3.626) | 32.600–50.805 | 0.9713 |

**Table S9.** Observed incidence-based richness, Chao2 estimates, 95% confidence intervals, and sample coverage for the unfiltered floristic matrix. Site-level uncertainty is wider where fewer analytical subplots were available.
